# Smc3 Deacetylation by Hos1 Facilitates Efficient Dissolution of Sister Chromatid Cohesion during Early Anaphase

**DOI:** 10.1016/j.molcel.2020.04.036

**Published:** 2020-05-21

**Authors:** Shuyu Li, Zuojun Yue, Tomoyuki U. Tanaka

(Molecular Cell *68*, 605–614.e1–e4; November 2, 2017)

The authors report an inadvertent error in Figure 2A. The unit of time on the y axis of the graph was mislabeled as “min.” The correct unit is “second.” The correct Figure 2A is shown below. The error does not change the conclusions of the figure. The authors apologize for any inconvenience the error may have caused.

**Figure 2A:**
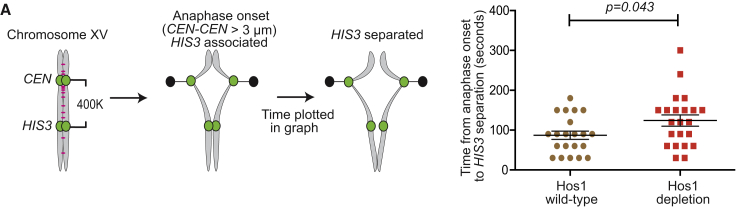
Separation of Sister *HIS3* Loci after Anaphase Onset Is Delayed in Hos1-Depleted Cells

