# Supplementary material for: Smc3 Deacetylation by Hos1 Facilitates Efficient Dissolution of Sister Chromatid Cohesion during Early Anaphase
Source: Mol Cell. 2017 Nov 2;68(3):605–614.e4. doi: 10.1016/j.molcel.2017.10.009 (PMC5678280; doi:10.1016/j.molcel.2017.10.009)
Supplement: Document S1. Figures S1–S4 and Table S1 [file mmc1.pdf]

Molecular Cell, Volume *68*

Supplemental Information

Smc3 Deacetylation by Hos1 Facilitates Efficient Dissolution of Sister Chromatid Cohesion during Early Anaphase

Shuyu Li, Zuojun Yue, and Tomoyuki U. Tanaka

Supplemental Information

Smc3 deacetylation by Hos1 facilitates efficient dissolution of sister chromatid cohesion during early anaphase

Shuyu Li, Zuojun Yue and Tomoyuki U. Tanaka

Centre for Gene Regulation and Expression, School of Life Sciences, University of Dundee, Dundee DD1 5EH, UK

Figure S1

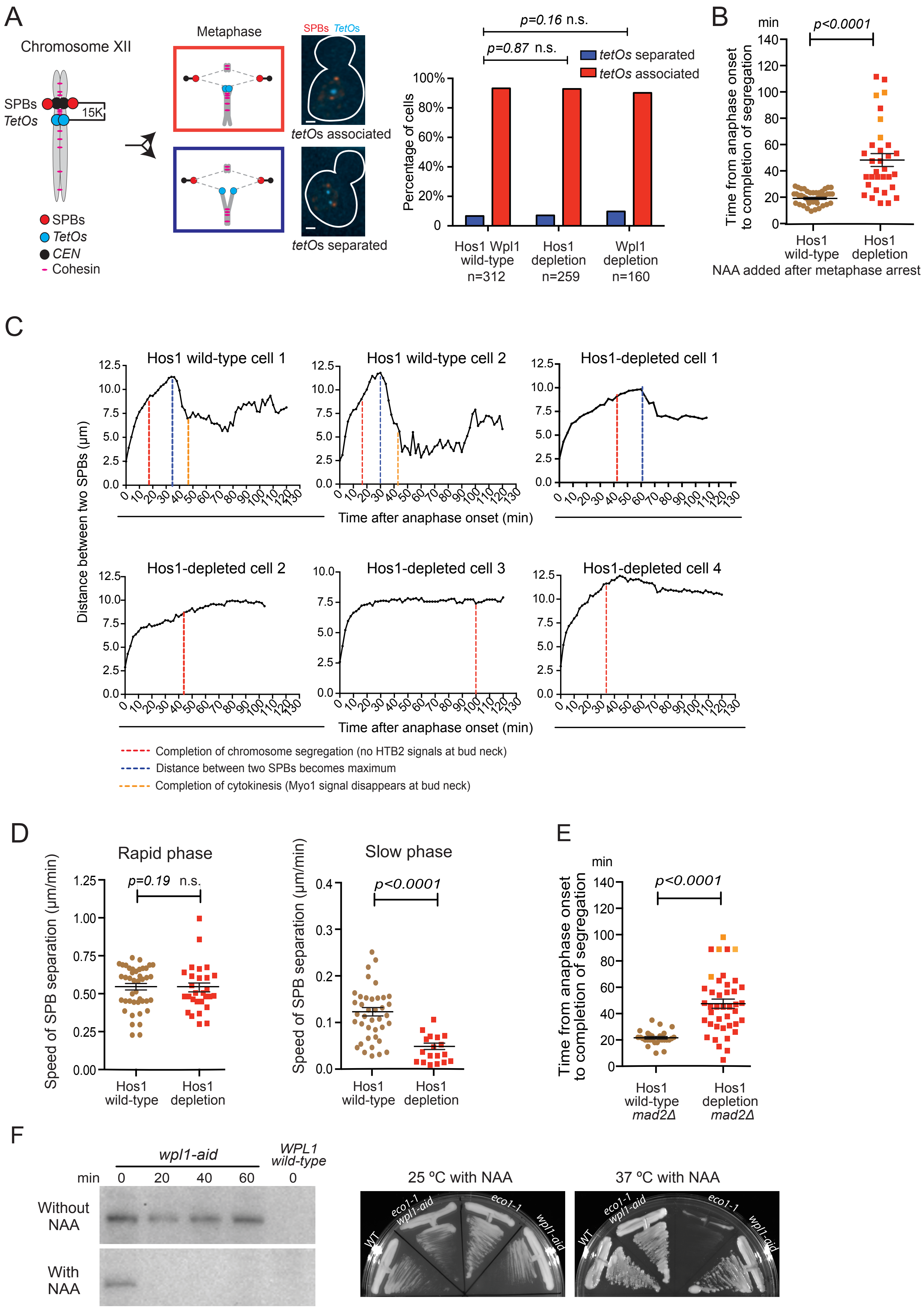
A
Chromosome XII
SPBs
TetOs
15K
SPBs
TetOs
CEN
Cohesin
Metaphase
SPBs TetOs
tetOs associated
tetOs separated
p=0.16 n.s.
p=0.87 n.s.
tetOs separated
tetOs associated
Percentage of cells
Hos1 Wpl1 wild-type n=312
Hos1 depletion n=259
Wpl1 depletion n=160
B
min
p<0.0001
Time from anaphase onset to completion of segregation
Hos1 wild-type
Hos1 depletion
NAA added after metaphase arrest
C
Hos1 wild-type cell 1
Hos1 wild-type cell 2
Hos1-depleted cell 1
Hos1-depleted cell 2
Hos1-depleted cell 3
Hos1-depleted cell 4
Distance between two SPBs (μm)
Time after anaphase onset (min)
Completion of chromosome segregation (no HTB2 signals at bud neck)
Distance between two SPBs becomes maximum
Completion of cytokinesis (Myo1 signal disappears at bud neck)
D
Rapid phase
p=0.19 n.s.
Speed of SPB separation (μm/min)
Hos1 wild-type
Hos1 depletion
Slow phase
p<0.0001
Speed of SPB separation (μm/min)
Hos1 wild-type
Hos1 depletion
E
min
p<0.0001
Time from anaphase onset to completion of segregation
Hos1 wild-type mad2Δ
Hos1 depletion mad2Δ
F
wpl1-aid
WPL1 wild-type
min 0 20 40 60 0
Without NAA
With NAA
25 °C with NAA
37 °C with NAA
WT
eco1-1 wpl1-aid
eco1-1
wpl1-aid

Figure S1 (related to Figure 1)

A) After depletion of Hos1 and Wpl1, sister chromatid cohesion at peri-centromere regions is maintained as robustly as in wild-type cells
Premature sister chromatid separation around the centromere (> 10 kb from *CEN*) in metaphase is indicative of a defect in sister chromatid cohesion. We addressed whether Hos1- and Wpl-depleted cells show a defect in sister chromatid cohesion. *HOS1 WPL1* wild-type (T11773), *hos1-aid* (T11814) and *wpl1-aid* (T11804) cells with *SPC42-mCherry*, P_{MET3}-*CDC20*, TetR-*3xCFP* and *tetO*s integrated at 15 kb away from *CEN12* on chromosome XII, were arrested in G1 phase with mating pheromone in methionine-dropout medium, and subsequently released to fresh YPA medium containing 2 mM additional methionine (to deplete Cdc20) and 0.5 mM NAA (to deplete Hos1-aid and Wpl1-aid). At 2 h after release from G1, the association and separation of sister CFP dots were scored in metaphase-arrested cells (distance between two SPBs < 2 μm).
There was no significant difference in the percentage of CFP-dot separation between wild-type, Hos1- and Wpl-depleted cells. This suggests that sister chromatid cohesion remains robust in metaphase in both Hos1- and Wpl1-depleted cells.

B) Chromosome segregation in anaphase was delayed after Hos1 was depleted during metaphase arrest
HOS1 wild-type (T11556) and *hos1-aid* (T11552) cells with *HTB2-CFP*, *SPC42-mCherry*, *MYO1-mCherry* and P_{MET3}-*CDC20*, were treated with mating pheromone to arrest in G1, and released to fresh YPA medium with 2 mM additional methionine to deplete Cdc20 and to arrest in metaphase. After 1 h following release from G1, NAA was added to deplete Hos1-aid. One hour after addition of NAA, cells were transferred to methionine dropout media with NAA to release them from metaphase arrest and allow them to progress to anaphase. Subsequently time-lapse images were taken every 2 min for 2 h. Time from anaphase onset to completion of chromosome segregation was measured as in Figure 1C. Orange squares in Hos1 depletion show the time when time-lapse observation finished without chromosome segregation being completed. *p* values were obtained by *t*-test.

C) Kinetics of spindle elongation during anaphase in wild-type and Hos1-depleted cell
HOS1 wild-type (T11219) and *hos1-aid* (T11218) cells with *HTB2-CFP*, *SPC42-mCherry*, and *MYO1-mCherry* were treated and their images acquired, as in Figure 1C. The length of the spindle, i.e. the distance between two spindle pole bodies (SPBs) was measured during anaphase in representative Hos1 wild-type and Hos1-depleted cells. The anaphase onset (time 0 in graphs) is defined as the time when the distance between two SPBs reached > 2.5 μm. Red dashed lines indicate the time of completion of chromosome segregation (defined as in Figure 1C). Blue dashed lines show the time when two SPBs reached the maximum separation, and the orange dashed lines indicate the time of completion of cytokinesis (disappearance of a Myo1 ring at the bud neck; Wloka and Bi 2012).

D) Comparison of spindle elongation speed during anaphase in wild-type and Hos1-depleted cell
The image sequences acquired in C were analyzed further. The speed of SPB separation in anaphase was calculated by dividing the change in SPB–SPB distance by the time spent in a rapid phase (SPB–SPB distance changing from 2.5 to 6.5 μm) and in a slow phase (from 6.5 μm to maximum distance). *p* values were obtained by *t*-test.

E) Spindle assembly checkpoint is not involved in the delay of chromosome segregation in Hos1-depleted cells
HOS1 wild-type (T11639) and *hos1-aid* (T11640) cells with *mad2Δ*, *HTB2-CFP*, *SPC42-mCherry* and *MYO1-mCherry* were treated and their images were acquired as in Figure 1C. The time from the anaphase onset to completion of chromosome segregation was measured as in Figure 1C. *p* value was obtained by *t*-test.

F) Wpl1 was rapidly degraded and its function was abolished by the auxin-induced degron system

Left: *WPL1* wild-type (T9855) and *wpl1-aid* (T11210) cells were incubated with or without 0.5 mM NAA in asynchronous culture, and analyzed after 20, 40 and 60 min by western blotting. Wpl1-aid protein was detected with an anti-AID tag antibody. The result shows that the majority of Wpl1-aid protein was degraded within 20 min following addition of NAA.

Right: *WPL1* wild-type (T9855), *wpl-aid eco1-1* (T11815), *eco1-1* (T4107) and *wpl1-aid* (T11210) cells were inoculated on YPAD plates containing 0.5 mM NAA and grown at 25°C and 37°C for two days. After addition of auxin NAA, *wpl1-aid* suppressed the growth defects of the *eco1-1* mutant at 37°C, as did the *wpl1* deletion (Rolef Ben-Shahar et al., 2008). This suggests that Wpl1 function was abolished by the auxin-induced degron system.

Figure S2

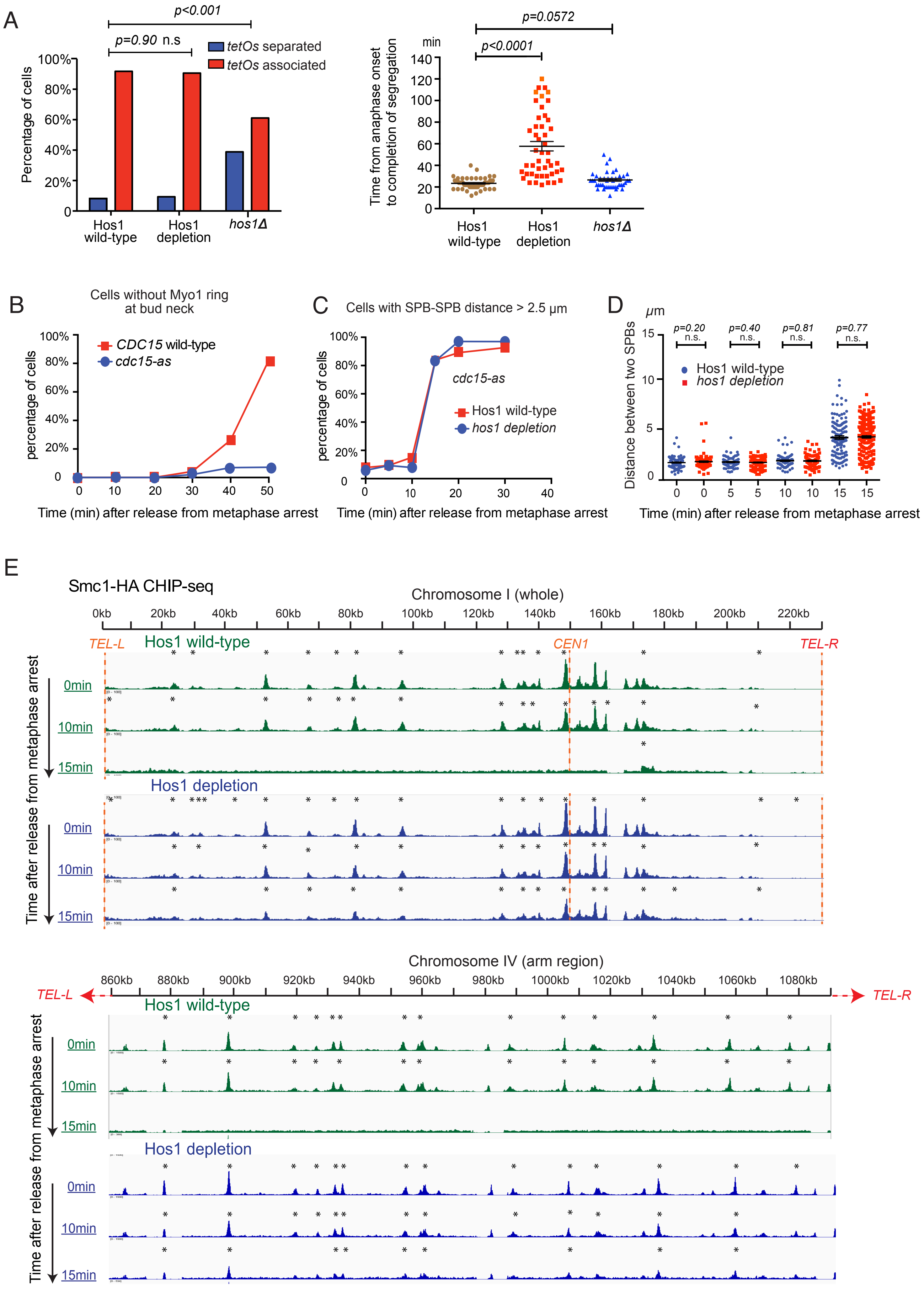

A
p<0.001
p=0.90 n.s
tetOs separated
tetOs associated
Percentage of cells
100%
80%
60%
40%
20%
0
Hos1 wild-type
Hos1 depletion
hos1Δ
p=0.0572
p<0.0001
min
Time from anaphase onset to completion of segregation
140
120
100
80
60
40
20
0
Hos1 wild-type
Hos1 depletion
hos1Δ
B
Cells without Myo1 ring at bud neck
CDC15 wild-type
cdc15-as
percentage of cells
Time (min) after release from metaphase arrest
C
Cells with SPB-SPB distance > 2.5 μm
cdc15-as
Hos1 wild-type
hos1 depletion
percentage of cells
Time (min) after release from metaphase arrest
D
μm
p=0.20 n.s.
p=0.40 n.s.
p=0.81 n.s.
p=0.77 n.s.
Hos1 wild-type
hos1 depletion
Distance between two SPBs
Time (min) after release from metaphase arrest
E
Smc1-HA CHIP-seq
Chromosome I (whole)
0kb
20kb
40kb
60kb
80kb
100kb
120kb
140kb
160kb
180kb
200kb
220kb
TEL-L
Hos1 wild-type
CEN1
TEL-R
0min
10min
15min
Hos1 depletion
0min
10min
15min
Time after release from metaphase arrest
Chromosome IV (arm region)
860kb
880kb
900kb
920kb
940kb
960kb
980kb
1000kb
1020kb
1040kb
1060kb
1080kb
TEL-L
TEL-R
Hos1 wild-type
0min
10min
15min
Hos1 depletion
0min
10min
15min
Time after release from metaphase arrest

Figure S2 (related to Figure 2)

A) Comparison of defects between *hos1* gene deletion (*hos1*Δ) and fresh Hos1 protein depletion

Cells with *HOS1* wild-type (T11773), *hos1-aid* (T11814) and *hos1*Δ (T13096) with *SPC42-mCherry*, P_{MET3}-*CDC20*, *TetR-3xCFP* and *tetO*s integrated at 15 kb away from *CEN12* on chromosome XII, were analyzed as in Figure S1A to compare strength of sister chromatid cohesion (left). The result suggests that, in contrast to fresh Hos1 depletion in the present cell cycle, *hos1*Δ cells show weakened cohesion, which is consistent with previous reports (Beckouet et al., 2010; Borges et al., 2010). It is thought that Smc3 is not re-cycled for the next cycle with *hos1*Δ. By contrast, cells should not have a defect in Smc3 recycling, when Hos1 is depleted following G1 phase and analyses are carried out in subsequent metaphase/anaphase. Meanwhile, in cells with *HOS1* wild-type (T11219), *hos1-aid* (T11218) and *hos1*Δ (T13109) with *HTB2-CFP*, *SPC42-mCherry*, and *MYO1-mCherry,* timing of completing chromosome segregation was analyzed as in Figure 1C (right). The result suggests that, in contrast to fresh Hos1 depletion in the present cell cycle, *hos1*Δ cells show only a marginal delay in completing chromosome segregation. We reason that, with *hos1*Δ*,* weaker cohesion in metaphase offsets a delay in cohesion removal in anaphase.

B) Inactivation of Cdc15-as kinase by an ATP analog prevents cells from completing cytokinesis

We tried to confirm that inhibiting Cdc15-as kinase by an ATP analog prevents cells from completing cytokinesis and entering the next cell cycle, in our experimental condition. *CDC15* wild-type (T12997) and *cdc15-as* (T11744) cells with *SMC1-HA, HTB2-CFP*, *SPC42-mCherry*, *MYO1-mCherry* and P_{GAL}-*CDC20* were treated, as in Figure 2C (NAA was not used). Cells were collected at 0, 10, 20, 30, 40, and 50 minutes, following release to anaphase, and fixed with 4% paraformaldehyde. At each time point, the percentage of cells without Myo1-ring signals at the bud neck was scored. Myo1 disappearance at the bud neck is an indicator of completed cytokinesis (Wloka and Bi, 2012). The result indicates that, when Cdc15-as kinase was inactivated by an ATP analog 1NM-PP1, most cells failed to complete cytokinesis.

C, D) Hos1 wild-type and Hos1-depleted cells start spindle elongation at similar timing in anaphase

HOS1 wild-type (T11875) and *hos1-aid* (T11874) cells with *cdc15-as*, *SMC1-HA*, *SPC42-mCherry, MYO1-mCherry* and P_{GAL}-*CDC20*, were treated and their images were acquired, as in Figure 2C. The SPB–SPB distance was measured in individual cells (C), and the percentage of cells with SPB–SPB distance > 2.5 μm was scored (B). Bars in C represent the mean and SE. *n.s.*: no significant difference in *t*-test.

E) ChIP-seq analysis of Smc1 localization on chromosomes.

HOS1 wild-type (T11875) and *hos1-aid* (T11874) cells (see C, D) were treated as in Figure 2C. Cells were collected at 0, 10, and 15 minutes after release to anaphase, and fixed with 1% formaldehyde. Chromatin immuno-precipitation with Smc1-HA was performed as described in STAR Methods. Immuno-precipitated DNA was analyzed using high-throughput DNA sequencing (ChIP-seq). The distribution of immuno-precipitated DNA is shown along chromosome I (whole) and chromosome IV (arm region). *TEL-L* and *TEL-R* represent the left and right telomere, respectively. Peaks in ChIP-seq were identified using MACS (Feng et al., 2012) (see STAR Methods) and those with length > 600 bp and enrichment >2.0 are highlighted by asterisks above the peaks.

Figure S3

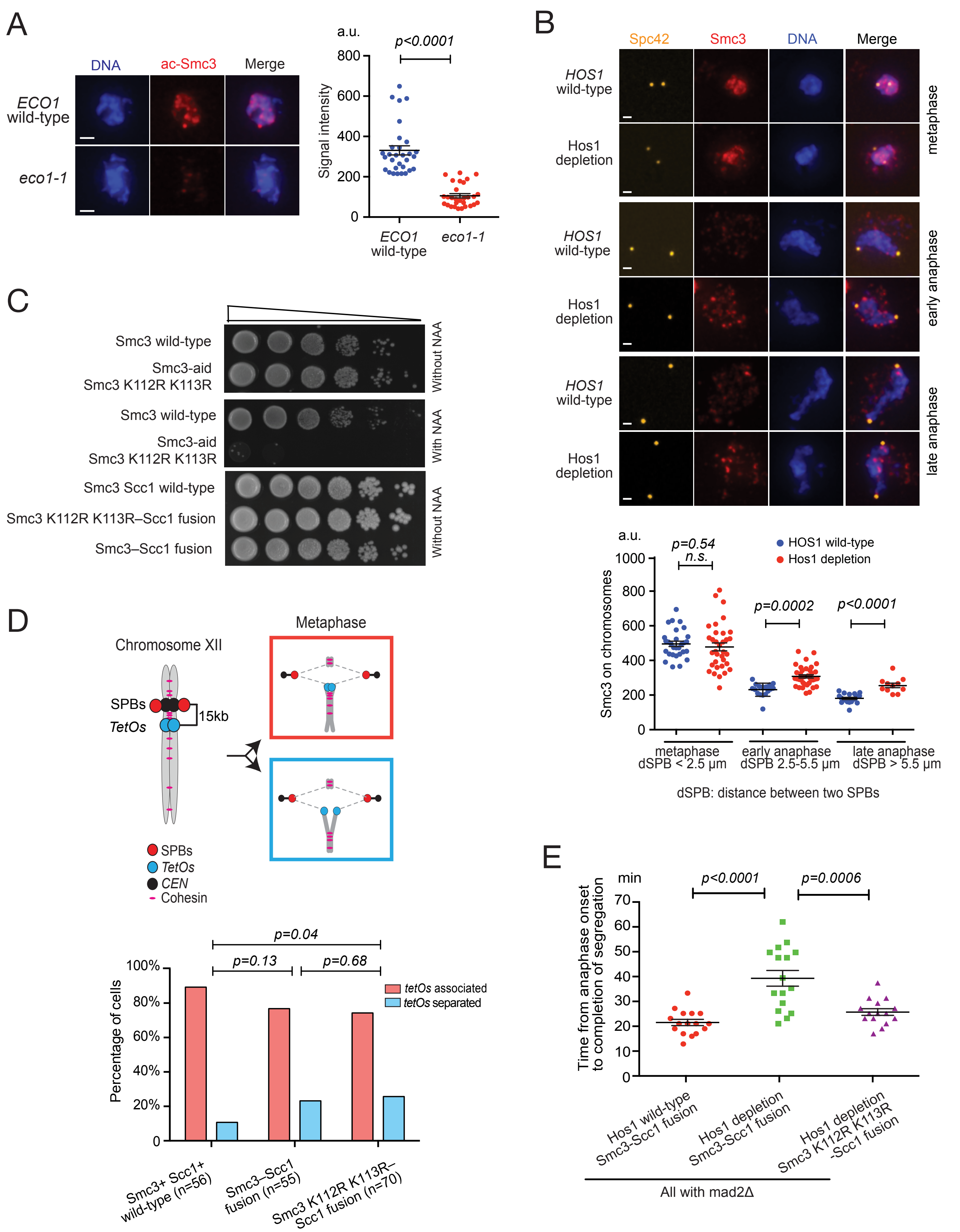

A
DNA
ac-Smc3
Merge
ECO1
wild-type
eco1-1
a.u.
p<0.0001
Signal intensity
800
600
400
200
0
ECO1
wild-type
eco1-1
B
Spc42
Smc3
DNA
Merge
HOS1
wild-type
Hos1
depletion
metaphase
early anaphase
late anaphase
a.u.
1000
800
600
400
200
0
p=0.54
n.s.
p=0.0002
p<0.0001
HOS1 wild-type
Hos1 depletion
Smc3 on chromosomes
metaphase
dSPB < 2.5 μm
early anaphase
dSPB 2.5-5.5 μm
late anaphase
dSPB > 5.5 μm
dSPB: distance between two SPBs
C
Smc3 wild-type
Smc3-aid
Smc3 K112R K113R
Without NAA
With NAA
Smc3 Scc1 wild-type
Smc3 K112R K113R–Scc1 fusion
Smc3–Scc1 fusion
D
Chromosome XII
SPBs
TetOs
15kb
Metaphase
SPBs
TetOs
CEN
Cohesin
p=0.04
p=0.13
p=0.68
100%
80%
60%
40%
20%
0
Percentage of cells
tetOs associated
tetOs separated
Smc3+ Scc1+
wild-type (n=56)
Smc3–Scc1
fusion (n=55)
Smc3 K112R K113R–
Scc1 fusion (n=70)
E
min
p<0.0001
p=0.0006
70
60
50
40
30
20
10
0
Time from anaphase onset
to completion of segregation
Hos1 wild-type
Smc3-Scc1 fusion
Hos1 depletion
Smc3-Scc1 fusion
Hos1 depletion
Smc3 K112R K113R
-Scc1 fusion
All with mad2Δ

Figure S3 (related to Figure 3)

A) Confirmation that a mouse anti-acetyl-Smc3 antibody can detect acetylated Smc3 on fixed and immobilized chromosomes.
ECO1 wild-type (K699) and *eco1-1* (K9435) cells were arrested in G1 phase with mating pheromone at 25°C. Then cells were incubated at 35°C for the last 30 min during G1 arrest and subsequently released from G1 arrest at 35°C by washing out mating pheromone. Cells were collected 60 min after the release. Chromosomes were fixed and immobilized on a slide glass immediately after cell lysis, followed by immunostaining of acetylated Smc3 (ac-Smc3). Scale bars represent 1 μm. *p* value was obtained by *t*-test. Bars and error bars show means and SEMs. a.u., arbitrary unit.

B) A larger amount of Smc3 remains on anaphase chromosomes in Hos1-depleted cells.
HOS1 wild type (T13179) and *hos1-aid* (T13180) cells with *SMC3-HA cdc20Δ* P_{GAL}*-CDC20 Spc42-mCherry* were treated as in Figure 3A. Chromosomes were fixed and immobilized as in Figure 3A, and Smc3-HA was stained using an anti-HA antibody. Representative cells are shown on top. The Smc3-HA signals on chromosomes were quantified (bottom). Cells in metaphase, in early anaphase and in late anaphase were defined as in Figure 3A. Scale bars represent 1 μm. *p* value was obtained by *t*-test. Bars and error bars show means and SEMs. *n.s.*, no significant difference. a.u., arbitrary unit.

C) Non-acetyl Smc3–Scc1 fusion (Smc3 K112R K113R–Scc1) can maintain cell viability in the absence of the original Smc3 and Scc1.
Ten-fold serial dilution of overnight cultures of *SMC3* wild-type (K700) and *smc3-aid smc3-K112R K113R* cells (T12989) cells were incubated for three days in the absence (top, left) and presence of (top, right) of NAA. *SMC3 SCC1* wild-type (K700), *SMC3 K112R K113R–SCC1* fusion (T12682) and *SMC3–SCC1* fusion (T12530) cells were also analyzed in the same way in the absence of NAA. In T12682 and T12530 cells, the original *SMC3* and *SCC1* genes were deleted.

D) Cells expressing non-acetyl and 'wild-type' Smc3–Scc1 fusion can similarly establish and maintain cohesion.
SMC3 SCC1 wild-type (T9968), *SMC3–SCC1* fusion (T12636) and *SMC3 K112R K113R–SCC1* fusion (T12638) cells with *SPC42-mCherry*, *TetR-GFP* and *tetO*s at 15 kb from *CEN12* on chromosome XII, were arrested in G1 phase with mating pheromone, released to fresh media and fixed at 70 min after the release. More than 70% cells were in metaphase, i.e. the distance between two SPBs was 1.5 –2.5 μm. In these cells, association and separation of sister *CEN* dots were scored (graph). *p* values were obtained by Fisher's exact test. In T12636 and T12638 cells, the original *SMC3* and *SCC1* genes were deleted.

E) Non-acetyl Smc3 mutant rescues timely chromosome segregation in Hos1-depleted cells, independently of spindle assembly checkpoint
HOS1 wild-type SMC3–SCC1 fusion (T12730), *hos1-aid SMC3–SCC1* fusion (T12729) and *hos1-aid SMC3-K112R K113R–SCC1* fusion (T12758) cells with *mad2*Δ, *SPC42-mCherry*, *MYO1-mCherry* and *HTB2-CFP*, were treated and their images acquired, as in Figure 3B. The time from the onset anaphase to completion of chromosome segregation was analyzed as in Figure 3B. The result confirms that non-acetyl Smc3-Scc1 fusion alleviates a delay in chromosome segregation in Hos1-depleted cells, and suggests that this effect is independent of spindle assembly checkpoint (in which Mad2 is a main regulator).

Figure S4

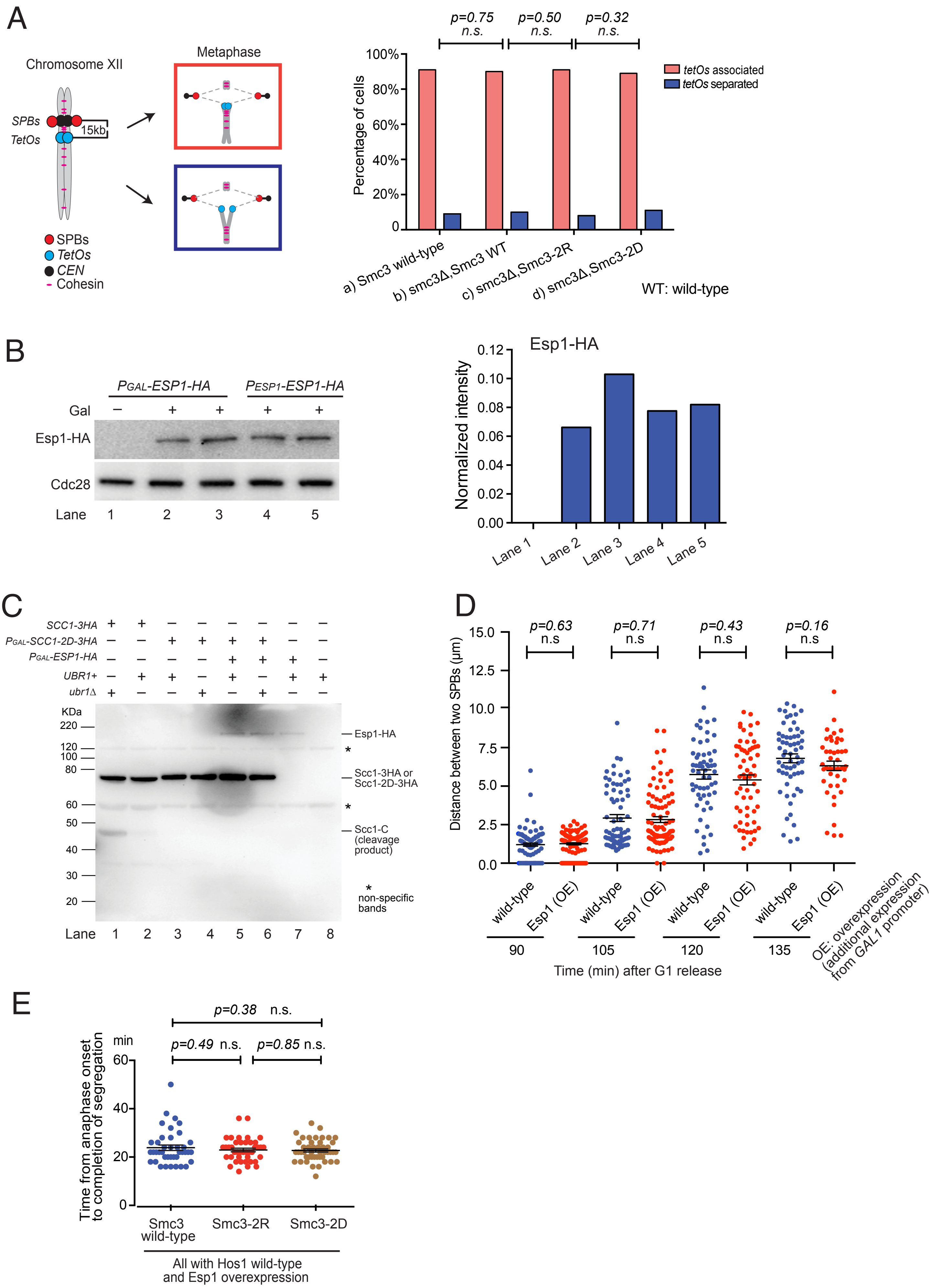
A
Chromosome XII
SPBs
TetOs
15kb
Metaphase
SPBs
TetOs
CEN
Cohesin
p=0.75
n.s.
p=0.50
n.s.
p=0.32
n.s.
100%
80%
60%
40%
20%
0
Percentage of cells
tetOs associated
tetOs separated
a) Smc3 wild-type
b) smc3Δ,Smc3 WT
c) smc3Δ,Smc3-2R
d) smc3Δ,Smc3-2D
WT: wild-type
B
PGAL-ESP1-HA
PESP1-ESP1-HA
Gal
Esp1-HA
Cdc28
Lane 1 2 3 4 5
Esp1-HA
Normalized intensity
0.12
0.10
0.08
0.06
0.04
0.02
0.00
Lane 1
Lane 2
Lane 3
Lane 4
Lane 5
C
SCC1-3HA
PGAL-SCC1-2D-3HA
PGAL-ESP1-HA
UBR1+
ubr1Δ
KDa
220
120
100
80
60
50
40
30
20
Esp1-HA
Scc1-3HA or
Scc1-2D-3HA
Scc1-C
(cleavage
product)
*
non-specific
bands
Lane 1 2 3 4 5 6 7 8
D
p=0.63
n.s
p=0.71
n.s
p=0.43
n.s
p=0.16
n.s
Distance between two SPBs (μm)
15.0
12.5
10.0
7.5
5.0
2.5
0.0
wild-type
Esp1 (OE)
90
105
120
135
Time (min) after G1 release
OE: overexpression
(additional expression
from GAL1 promoter)
E
p=0.38 n.s.
p=0.49 n.s.
p=0.85 n.s.
min
60
40
20
0
Time from anaphase onset
to completion of segregation
Smc3
wild-type
Smc3-2R
Smc3-2D
All with Hos1 wild-type
and Esp1 overexpression

Figure S4 (related to Figure 4)

A) An *smc3* mutant, which can be cleaved by separase (*SMC3-2R*), and its control (*SMC3-2D*) can establish and maintain robust sister chromatid cohesion
We addressed whether *SMC3-2R* and *-2D* mutants can establish and maintain sister chromatid cohesion, similarly to *SMC3* wild-type (T12623; *b* in graph). *SMC3-2R* (T12249; *c*) and *SMC3-2D* (T12255; *d*) cells with with *SPC42-mCherry*, P_{MET3}-*CDC20*, *TetR-3xCFP* and *tetO*s integrated at 15 kb away from *CEN12* on chromosome XII, were treated and analyzed as in Figure S1A. In these cells, *SMC3* was deleted at the original locus whereas *SMC3* wild-type and mutant constructs were integrated at an auxotroph locus. Control cells with intact *SMC3* locus (T11773; *a* in graph) were also analyzed in the same way. Number of cells analyzed was 165, 245, 268 and 305 for *a*, *b*, *c* and *d*, respectively. The percentage of cells with two sister CFP dots was not significantly different among the four strains. This result indicates that *SMC3-2R* and *-2D* mutants can maintain normal robust sister chromatid cohesion in metaphase.

B) The *GAL1* and *ESP1* promoters support a similar level of separase (Esp1) expression
Cells with P_{GAL}-*ESP1-HA* (T13101) and P_{ESP1}-*ESP1-HA* (T13102), in both of which *ESP1* was fused a single copy of the *HA* tag, were cultured with (lane 2–5) and without (lane 1) galactose for 2.5 h. P_{GAL} and P_{ESP1} represent the *GAL1* and *ESP1* promoter, respectively. Lanes 2 and 3 as well as lanes 4 and 5 show the outcomes from two independent cultures of the same strain in the same culture conditions. In T13101, P_{GAL}-*ESP1-HA* was inserted at an auxotroph locus while the original *ESP1* locus was intact. In T13102, HA was fused with the *ESP1* gene at its original locus. They were analyzed by a western blot, using anti-HA and anti-Cdc28 antibodies (left). The intensity of Esp1-HA was normalized to the intensity of Cdc28 (right). The result suggests the *GAL1* and *ESP1* promoters support a similar level of Esp1 expression. In other words, in cells with P_{GAL}-*ESP1* (with the original *ESP1* locus intact), the level of Esp1 approximately doubled in the presence of galactose in medium.

C) With separase (Esp1) overexpression, Scc1-2D still does not show cleavage.
Cells with *SCC1-3HA ubr1Δ* (T13221, lane 1) and *SCC1-3HA* (T4120, lane 2) were cultured in YPAD medium. Cells with P_{GAL}-*SCC1-2D-3HA SMC3-2D* (T12201, lane 3), P_{GAL}-*SCC1-2D-3HA ubr1Δ SMC3-2D* (T13231, lane 4), P_{GAL}-*SCC1-2D-3HA* P_{GAL}-*ESP1-HA SMC3-2D* (T13210, lane 5), P_{GAL}-*SCC1-2D-3HA* P_{GAL}-*ESP1-HA ubr1Δ SMC3-2D* (T13220, lane 6) and P_{GAL}-*ESP1-HA* (T12893, lane 7) were cultured in YPA-raffinose medium overnight and subsequently in YPA-raffinose-galactose media for 2.5 h. Strain (K699) without any HA-tagged genes was used as a control (lane 8). Cells were harvested from asynchronous culture and analyzed by a western blot using an anti-HA antibody. We could detect Scc1 cleavage products when *ubr1* gene was deleted (without the N-end rule pathway; lane 1). However we could not detect cleavage products of Scc1-2D even when *ubr1* was deleted, with or without Esp1 overexpression (lanes 4 and 6).

D) With separase (Esp1) overexpression, the spindle elongation still occurs with normal kinetics during anaphase.
ESP1 wild-type cells (T12258) and cells carrying P_{GAL}-*ESP1* (T12565), both with *SPC42-mCherry, MYO1-mCherry, HTB2-CFP, hos1-aid* and *SMC3-2D*, were arrested in G1 phase with mating pheromone in YPA medium with raffinose. Cells were released to fresh YPA medium containing raffinose. Galactose was added for the last 30 min during G1 arrest and during the subsequent release from G1 (to overexpress Esp1 in T12565 cells). At indicated time points, cells were collected and fixed with 4% paraformaldehyde. The distance between two SPBs was measured in individual cells and plotted. Bars show mean and SEM. *n.s.*: no significant difference in *t*-test. The result suggests there was no precocious cohesin cleavage when Esp1 was overexpressed in this condition; if there had been precocious cohesin cleavage, we would have observed earlier spindle elongation.
Note that, with separase overexpression, we did not detect a significant change in the amount of Smc3-2R cleavage products (Figure 4B, lane 5), but Smc3-2R did show a much higher rate of chromosome segregation in the presence of Scc1-2D (Figure 4C, magenta

bars). This is explained if fresh Smc3-2R cleavage is more efficient in the presence of separase overexpression, while Smc3 cleavage products in previous cycles may remain and be detected in Figure 4B.

E) *SMC3-2R* and *SMC3-2D* do not change timing of completing chromosome segregation in Hos1 wild-type cells.

SMC3 wild-type (12986), *SMC3-2R* (T13225) and *SMC3-2D* (T13226) cells with P_{GAL}-*ESP1*, *HTB2-CFP*, *SPC42-mCherry* and *MYO1-mCherry* were arrested in G1 phase with mating pheromone in YPA-raffinose media, and released to fresh media. 2% galactose was added for the last 30 min during G1 arrest and subsequent release from G1. Images were acquired as in Figure 1C. Time from the anaphase onset to completion of chromosome segregation was measured and plotted in individual cells, as in Figure 1C.

Table S1 (related to STAR Methods)

The table shows genotypes of yeast strains used in this study. All strains used in this study are derivatives of *Saccharomyces cerevisiae* W303 (K699 and K700 from K. Nasmyth lab).

Name	Genotype
K699	*MATa ade2-1 trp1-1 leu2-3,112 his3-11,15 ura3 can1-100*
K700	*MATα ade2-1 trp1-1 leu2-3,112 his3-11,15 ura3 can1-100*
K9435	*MATα eco1-1*
T4107	*MAT α eco1-1, POL1-4×GFP::kanMX*
T4120	*MATa SCC1-3HA::HIS3 smc3Δ::HIS3 leu2::SMC3-TEVsite::LEU2 trp1::*P_{GAL}*-TEV::TRP1*
T9968	*MATa ade1::tetR-3×CFP::hphN1 SPC42-4×mCherry::natMX6 NIC96-4×mCherry::natMX6 tetO224 (LEU2) is integrated at +15kb from CEN12*
T9855	MAT α *ura3::*P_{ADH1}*-osTIR1-9×myc::URA3*
T10829	*MATa leu2::TetR-GFP::LEU2 ChrXV 326K::tetO112::kanMX ChrXV his3::tetO112::HIS3 ChrXV 1070K::tetO112::HphN1 hos1-3×mini-aid::kanMX ura3::*P_{ADH1}*-osTIR1-9×myc::URA3*
T10830	MATa *leu2::TetR-GFP::LEU2 ChrXV 326K::tetO112::kanMX ChrXV his3::tetO112::HIS3, ChrXV 1070K::tetO112::HphN1 ura3::*P_{ADH1}*-osTIR1-9×myc::URA3*
T10954	*MATa/ α SMC3/smc3 Δ ::kanMX*
T11112	*MATa leu2::TetR-GFP::LEU2 ChrXV 1070K::tetO112::HphN1 hos1-3×mini-aid::kanMX ura3::*P_{ADH1}*-osTIR1-9×myc::URA3 SPC42-mCherry::natNT2*
T11113	*MATa leu2::TetR-GFP::LEU2 ChrXV 1070K::tetO112::HphN1 SPC42-mCherry::natNT2*
T11116	*MAT α smc3Δ::KanMX leu2::SMC3-2R-6×myc::LEU2*
T11117	*MAT α smc3Δ::KanMX leu2::SMC3-2D-6×myc::LEU2*
T11210	*MAT α wpl1-3×mini-aid::kanMX ura3::*P_{ADH1}*-osTIR1-9×myc::URA3*
T11218	*MATa SPC42-mCherry::natNT2 MYO1-4×mCherry::natNT2 HTB2-CFP::spHIS5 hos1-3×mini-aid::kanMX ura3::*P_{ADH1}*-osTIR1-9×myc::URA3*
T11219	*MAT α SPC42-mCherry::natNT2 MYO1-4×mCherry::natNT2 HTB2-CFP::spHIS5 ura3::*P_{ADH1}*-osTIR1-9×myc::URA3*
T11432	*MATa wpl1-3×mini-aid::kanMX ura3::*P_{ADH1}*-osTIR1-9×myc::URA3 SPC42-mCherry::natNT2 MYO1-4×mCherry::natNT2 HTB2-CFP::spHIS5*
T11552	*MATa hos1-3×mini-aid::kanMX ura3::*P_{ADH1}*-osTIR1-9×myc::URA3 SPC42-mcherry::natNT2 MYO1-4×mCherry::natNT2 HTB2-CFP::SpHIS5,* P_{MET3}*-CDC20::TRP1*
T11556	*MATa ura3::*P_{ADH1}*-osTIR1-9×myc::URA3 SPC42-mCherry::natNT2 MYO1-4×mcherry::natNT2 HTB2-CFP::SpHIS5,* P_{MET3}*-CDC20::TRP1*
T11639	*MATa SPC42-mCherry::natNT2 MYO1-4×mCherry::natNT2 HTB2-CFP::spHIS5 ura3::*P_{ADH1}*-osTIR1-9×myc::URA3 mad2 Δ ::hphNT1*
T11640	*MATa SPC42-mCherry::natNT2 MYO1-4×mCherry::natNT2 HTB2-CFP::spHIS5 hos1-3×mini-aid::kanMX ura3::*P_{ADH1}*-osTIR1-9×myc::URA3 mad2 Δ ::hphNT1*
T11710	*MATa leu2::*P_{URA3}*-TetR-GFP::LEU2 CEN15::tetOs::kanMX, ade2::lacO256::ADE2 his3::tetOs::HIS3 trp1::*P_{CUP1}*-3×CFP-lacI-I12-NLS::TRP1 ura3::*P_{ADH1}*-osTIR1-9×myc::URA3*
T11713	*MATa leu2::*P_{URA3}*-TetR-GFP::LEU2 CEN15::tetOs::kanMX ade2::lacO256::ADE2 his3::tetOs::HIS3 trp1::*P_{CUP1}*-3×CFP-lacI-I12-NLS::TRP1 hos1-aid::KanMX ura3::*P_{ADH1}*-osTIR1-9×myc::URA3*
T11744	*MATa ura3::*P_{ADH1}*-osTIR1-9×myc::URA3 cdc20 Δ ::LEU2 trp1::* P_{GAL}*-CDC20::TRP1 SMC1-6×HA::HisMX MYO1-4×mCherry::natNT2 cdc15-as1(L99G)::URA3*
T11773	*MATa* P_{MET3}*-CDC20::TRP1 ade1::tetR-3×CFP::hphN1 SPC42-4×mCherry::natMX6 NIC96-4×mCherry::natMX6 ura3::*P_{ADH1}*-osTIR1-9×myc::URA3 tetO224 (LEU2) is integrated at +15-kb from CEN12*
T11804	*MATa* P_{MET3}*-CDC20::TRP1 ade1::tetR-3×CFP::hphN1 SPC42-4×mCherry::natMX6 NIC96-4×mCherry::natMX6 wpl1-3xmini-aid::kanMX ura3::*P_{ADH1}*-osTIR1-9×myc::URA3 tetO224 (LEU2) is integrated at +15-kb from CEN12*
T11815	*MAT α eco1-1 wpl1-3xmini-aid::kanMX ura3::*P_{ADH1}*-osTIR1-9×myc::URA3*
T11814	*MATa* P_{MET3}*-CDC20::TRP1 ade1::tetR-3×CFP::HPH1 SPC42-4×mCherry::natMX6 NIC96-4×mCherry::natMX6 hos1-3×mini-aid::kanMX ura3::*P_{ADH1}*-osTIR1-9×myc::URA3 tetO224 (LEU2) is integrated at +15kb from CEN12*
T11874	*MATa SPC42-mCherry::natNT2 MYO1-4×mCherry::natNT2 HTB2-CFP::spHIS5 hos1-3×mini-aid::kanMX ura3::*P_{ADH1}*-osTIR1-9×myc::URA3 cdc20 Δ ::LEU2 trp1::*P_{GAL}*-CDC20::TRP1 SMC1-6×HA::HisMX cdc15-as1(L99G)::URA3*
T11875	*MATa SPC42-mCherry::natNT2 MYO1-4×mCherry::natNT2 HTB2-CFP::spHIS5 ura3::*P_{ADH1}*-osTIR1-9×myc::URA3 cdc20 Δ ::LEU2 trp1::* P_{GAL}*-CDC20::TRP1 SMC1-6×HA::HisMX cdc15-as1(L99G)::URA3*
T11877	*MATa SPC42-mCherry::natNT2 MYO1-4×mCherry::natNT2 HTB2-CFP::spHIS5 ura3::*P_{ADH1}*-osTIR1-9×myc::URA3 cdc20 Δ ::LEU2 trp1::* P_{GAL}*-CDC20::TRP1 cdc15-as1(L99G)::URA3*
T11912	*MATa SPC42-mCherry::natNT2 MYO1-4×mCherry::natNT2 cdc20 Δ ::LEU2 trp1::GAL-CDC20::TRP1 cdc15-as1(L99G)::URA3 hos1-3×mini-aid::kanMX ura3::*P_{ADH1}*-osTIR1-*

	9×myc::URA3 SCC1-6×HA::hphN1
T11914	*MATa SPC42-mCherry::natNT2 MYO1-4×mCherry::natNT2 cdc20Δ::LEU2 trp1:: P_{GAL}-CDC20::TRP1 cdc15-as1(L99G)::URA3 ura3::P_{ADH1}-osTIR1-9×myc::URA3 SCC1-6×HA::hphN1*
T12200	*MATa ura3::P_{ADH1}-osTIR1-9×myc::URA3 SPC42-mCherry::natNT2 MYO1-4×mcherry::natNT2 HTB2-CFP::SpHIS5 smc3Δ::kanMX leu2::SMC3-2R-6×myc::LEU2 ade2::P_{GAL}-scc1 R180D R268D-3HA::ADE2*
T12201	*MATa ura3::P_{ADH1}-osTIR1-9×myc::URA3 SPC42-mCherry::natNT2 MYO1-4×mcherry::natNT2 HTB2-CFP::SpHIS5 smc3Δ::kanMX leu2::SMC3-2D-6×myc::LEU2 ade2::P_{GAL}-scc1 R180D R268D-3HA::ADE2*
T12249	*MATa P_{MET3}-CDC20::TRP1 ade1::tetR-3×CFP::hphN1 SPC42-4×mCherry::natMX6 NIC96-4×mCherry::natMX6 ura3::P_{ADH1}-osTIR1-9×myc::URA3 tetO224 (LEU2) is integrated at +15-kb from CEN12 smc3Δ::kanMX, leu2::SMC3-2R-6×myc::LEU2*
T12255	*MATa P_{MET3}-CDC20::TRP1 ade1::tetR-3×CFP::hphN1 SPC42-4×mCherry::natMX6 NIC96-4×mCherry::natMX6 ura3::P_{ADH1}-osTIR1-9×myc::URA3 tetO224 (LEU2) is integrated at +15kb from CEN12 smc3Δ::kanMX, leu2::SMC3-2D-6×myc::LEU2*
T12258	*MATa SPC42-mCherry::natNT2 MYO1-4×mCherry::natNT2 HTB2-CFP::spHIS5 hos1-3×mini-aid::kanMX ura3::P_{ADH1}-osTIR1-9×myc::URA3 smc3Δ::kanMX leu2::SMC3-2D-6×myc::LEU2*
T12426	*MATa/α SMC3/smc3Δ::kanMX SCC1/scc1Δ::natNT2*
T12530	*MATα smc3Δ::KanMX scc1Δ::natNT2 ura3::SMC3-SCC1::URA3 trp1::P_{ADH1}-osTIR1-9×myc::TRP1*
T12565	*MATa SPC42-mCherry::natNT2 MYO1-4×mCherry::natNT2 HTB2-CFP::spHIS5 hos1-3×mini-aid::kanMX ura3::P_{ADH1}-osTIR1-9×myc::URA3 smc3Δ::kanMX leu2::SMC3-2D-6×myc::LEU2 trp1::$P_{GAL1-10}$-ESP1-HA::TRP1*
T12566	*MATa SPC42-mCherry::natNT2 MYO1-4×mCherry::natNT2 HTB2-CFP::spHIS5 hos1-3×mini-aid::kanMX ura3::P_{ADH1}-osTIR1-9×myc::URA3 smc3Δ::KanMX leu2::SMC3-2R-6×myc::LEU2 trp1::$P_{GAL1-10}$-ESP1-HA::TRP1*
T12623	*MATa P_{MET3}-CDC20::TRP1 ade1::tetR-3×CFP::hphN1 SPC42-4×mCherry::natMX6 NIC96-4×mCherry::natMX6 ura3::P_{ADH1}-osTIR1-9×myc::URA3 tetO224 (LEU2) is integrated at +15-kb from CEN12 smc3Δ::kanMX, leu2::SMC3::LEU2*
T12636	*MATa ade1::tetR-3×CFP::hphN1 SPC42-4×mCherry::natMX6 NIC96-4×mCherry::natMX6 tetO224 (LEU2) is integrated at +15-kb from CEN12 smc3Δ::KanMX scc1Δ::natNT2 ura3::SMC3-SCC1::URA3*
T12638	*MATa ade1::tetR-3×CFP::hphN1 SPC42-4×mCherry::natMX6 NIC96-4×mCherry::natMX6 tetO224 (LEU2) is integrated at +15-kb from CEN12 smc3Δ::KanMX scc1Δ::natNT2 ura3::SMC3 K112R K113R-SCC1::URA3*
T12665	*MATa SPC42-mCherry::natNT2, MYO1-4×mcherry::natNT2 HTB2-CFP::SpHIS5 smc3Δ::KanMX scc1Δ::natNT2 ura3::SMC3-SCC1::URA3 hos1-aid::kanMX trp1::P_{ADH1}-osTIR1-9myc::TRP1*
T12666	*MATa SPC42-mCherry::natNT2, MYO1-4×mcherry::natNT2 HTB2-CFP::SpHIS5 smc3Δ::kanMX scc1Δ::natNT2 ura3::SMC3 K112R K113R-SCC1::URA3 hos1-aid::kanMX trp1::P_{ADH1}-osTIR1-9×myc::TRP1*
T12682	*MATα smc3Δ::kanMX scc1Δ::natNT2 ura3::SMC3 K112R K113R-SCC1::URA3 trp1::PADH1-osTIR1-9×myc::TRP1*
T12684	*MATa SPC42-mCherry::natNT2, MYO1-4×mcherry::natNT2 HTB2-CFP::SpHIS5 smc3Δ::KanMX scc1Δ::natNT2 ura3::SMC3-SCC1::URA3 trp1::P_{ADH1}-osTIR1-9×myc::TRP1*
T12724	*MATa MYO1-4×mCherry::natNT2 cdc20Δ::LEU2 trp1::P_{GAL}-CDC20::TRP1 cdc15-as1(L99G)::URA3 hos1-3×mini-aid::kanMX ura3::P_{ADH1}-osTIR1-9×myc::URA3 SCC1-6×HA::hphN1 ubr1-aid::kanMX*
T12726	*MATa MYO1-4×mCherry::natNT2 cdc20Δ::LEU2 trp1::P_{GAL}-CDC20::TRP1 cdc15-as1(L99G)::URA3 ura3::P_{ADH1}-osTIR1-9×myc::URA3 SCC1-6×HA::hphN1 ubr1-aid::kanMX*
T12729	*MATa SPC42-mCherry::natNT2, MYO1-4×mcherry::natNT2 HTB2-CFP::SpHIS5 smc3Δ::KanMX Scc1Δ::natNT2 ura3::SMC3-SCC1::URA3 trp1::P_{ADH1}-osTIR1-9×myc::TRP1 hos1-aid::kanMX mad2Δ::hphN1*
T12730	*MATa SPC42-mCherry::natNT2, MYO1-4×mcherry::natNT2 HTB2-CFP::SpHIS5 smc3Δ::KanMX Scc1Δ::natNT2 ura3::SMC3-SCC1::URA3 trp1::P_{ADH1}-osTIR1-9×myc::TRP1 mad2Δ::hphN1*
T12758	*MATa SPC42-mCherry::natNT2, MYO1-4×mcherry::natNT2 HTB2-CFP::SpHIS5 smc3Δ::kanMX Scc1Δ::natNT2 ura3::smc3 K112R K113R-SCC1::URA3 trp1::P_{ADH1}-osTIR1-9×myc::TRP1 hos1-aid::kanMX mad2Δ::hphN1*
T12825	*MATa ura3::P_{ADH1}-osTIR1-9×myc::URA3 SPC42-mCherry::natNT2 MYO1-4×mcherry::natNT2 HTB2-CFP::SpHIS5 smc3Δ::KanMX leu2::SMC3-2R-6×myc::LEU2 ade2::P_{GAL}-SCC1 R180D R268D-3HA::ADE2 trp1::$P_{GAL1-10}$-ESP1-HA::TRP1*
T12827	*MATa ura3::P_{ADH1}-osTIR1-9×myc::URA3 SPC42-mCherry::natNT2 MYO1-4×mcherry::natNT2 HTB2-CFP::SpHIS5 smc3Δ::kanMX leu2::SMC3-2D-6×myc::LEU2 ade2::P_{GAL}-SCC1 R180D R268D-3HA::ADE2 trp1::$P_{GAL1-10}$-*

	ESP1-HA::TRP1
T12892	*MAT α smc3Δ::KanMX leu2::SMC3-2R-6×myc::LEU2 trp1::*$P_{GAL1-10}$*-ESP1-HA::TRP1*
T12893	*MAT α smc3Δ::KanMX leu2::SMC3-2D-6×myc::LEU2 trp1::*$P_{GAL1-10}$*-ESP1-HA::TRP1*
T12975	*MAT α smc3Δ::KanMX leu2::SMC3-6×myc::LEU2*
T12976	*MAT α smc3Δ::KanMX leu2::SMC3-6×myc::LEU2 trp1::*$P_{GAL1-10}$*-ESP1-HA::TRP1*
T12986	*MATa ura3::*P_{ADH1}*-osTIR1-9×myc::URA3 SPC42-mCherry::natNT2 MYO1-4×mCherry::natNT2 HTB2-CFP::SpHIS5 trp1::*$P_{GAL1-10}$*-ESP1-HA::TRP1*
T12989	*MAT α smc3-3×mini-aid::KanMX ura3::*P_{ADH1}*-osTIR1-9×myc::URA3 leu2::SMC3 K112R K113R::LEU2*
T12997	*MATa ura3::*P_{ADH1}*-osTIR1-9×myc::URA3 cdc20 Δ ::LEU2 trp1::* P_{GAL}*-CDC20::TRP1 SMC1-6×HA::HisMX MYO1-4×mCherry::natNT2*
T13096	*MATα* P_{MET3}*-CDC20::TRP1 ade1::tetR-3×CFP::hphN1 SPC42-4×mCherry::natMX6 NIC96-4×mCherry::natMX6 ura3::*P_{ADH1}*-osTIR1-9×myc::URA3 tetO224 (LEU2) is integrated at +15kb from CEN12 hos1Δ::kanMX*
T13109	*MATa ura3::*P_{ADH1}*-osTIR1-9×myc::URA3 SPC42-mCherry::natNT2, MYO1-4×mCherry::natNT2 HTB2-CFP::SpHIS5 hos1Δ::kanMX*
T13101	*MATa trp1::*P_{GAL}*-ESP1-HA::TRP1*
T13102	*MATa ESP1-HA::kanMX*
T13179	*MATa SMC3-6×HA::hphN1 ura3::*P_{ADH1}*-osTIR1-9×myc::URA3 cdc20 Δ ::LEU2 trp1::*P_{GAL}*-CDC20::TRP1 SPC42-mCherry::natNT2*
T13180	*MATa SMC3-6×HA::hphN1 hos1-3×mini-aid::kanMX ura3::*P_{ADH1}*-osTIR1-9×myc::URA3 cdc20 Δ ::LEU2 trp1::*P_{GAL}*-CDC20::TRP1 SPC42-mCherry::natNT2*
T13221	*MATa SCC1-3HA::HIS3 ubr1Δ::hphN1 smc3Δ::HIS3 leu2::SMC3-TEVsite::LEU2 trp1::*P_{GAL}*-TEV::TRP1*
T13231	*MATa ura3::*P_{ADH1}*-osTIR1-9×myc::URA3 SPC42-mCherry::natNT2 MYO1-4×mcherry::natNT2 HTB2-CFP::SpHIS5 smc3Δ::kanMX leu2::SMC3-2D-6×myc::LEU2 ade2::*P_{GAL}*-SCC1 R180D R268D-3HA::ADE2*
T13210	*MAT α smc3Δ::KanMX leu2::SMC3-2D-6×myc::LEU2 trp1::*$P_{GAL1-10}$*-ESP1-HA::TRP1 ade2::*P_{GAL}*-SCC1 R180D R268D-3HA::ADE2*
T13220	*MAT α smc3Δ::KanMX leu2::SMC3-2D-6×myc::LEU2 trp1::*$P_{GAL1-10}$*-ESP1-HA::TRP1 ade2::*P_{GAL}*-SCC1 R180D R268D-3HA::ADE2 ubr1Δ::hphN1*
T13225	*MAT α ura3::*P_{ADH1}*-osTIR1-9×myc::URA3 SPC42-mCherry::natNT2, MYO1-4×mCherry::natNT2 HTB2-CFP::SpHIS5 smc3Δ::KanMX leu2::SMC3-2D-6×myc::LEU2 trp1::*$P_{GAL1-10}$*-ESP1-HA::TRP1*
T13226	*MAT α ura3::*P_{ADH1}*-osTIR1-9×myc::URA3 SPC42-mCherry::natNT2, MYO1-4×mcherry::natNT2 HTB2-CFP::SpHIS5 smc3Δ::KanMX leu2::SMC3-2R-6×myc::LEU2 trp1::*$P_{GAL1-10}$*-ESP1-HA::TRP1*
