# Supplementary material for: Smc3 Deacetylation by Hos1 Facilitates Efficient Dissolution of Sister Chromatid Cohesion during Early Anaphase
Source: Mol Cell. 2017 Nov 2;68(3):605–614.e4. doi: 10.1016/j.molcel.2017.10.009 (PMC5678280; doi:10.1016/j.molcel.2017.10.009)
Supplement: Document S2. Article plus Supplemental Information [file mmc7.pdf]

Short Article

Molecular Cell

Smc3 Deacetylation by Hos1 Facilitates Efficient Dissolution of Sister Chromatid Cohesion during Early Anaphase

Graphical Abstract

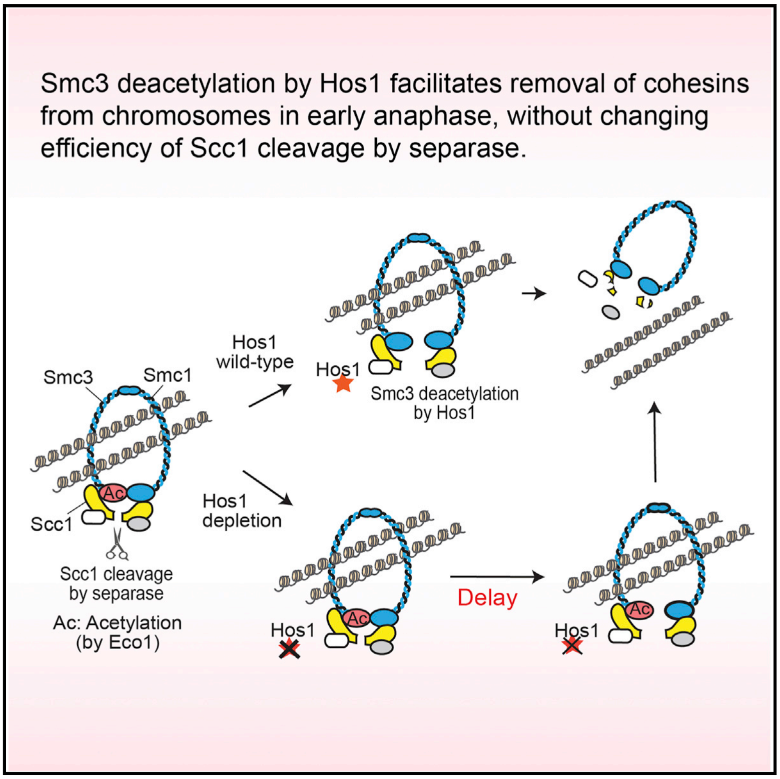

Authors

Shuyu Li, Zuojun Yue, Tomoyuki U. Tanaka

Correspondence

t.tanaka@dundee.ac.uk

In Brief

Sister chromatid cohesion relies on cohesins and is dissolved when cells enter anaphase. Li et al. show that, in addition to cleavage of cohesin Scc1 by separase, deacetylation of cohesin Smc3 by Hos1 is important for efficient removal of cohesins from chromosomes, which ensures timely chromosome segregation in mitosis.

Highlights

- Depletion of Hos1 causes a significant delay in completing chromosome segregation
- Smc3 deacetylation by Hos1 facilitates cohesin removal from chromosomes in anaphase
- Depletion of Hos1 does not change the efficiency of Scc1 cleavage by separase
- Smc3 deacetylation by Hos1 probably leads to disengagement of Smc1-Smc3 heads

Li et al., 2017, Molecular Cell *68*, 605–614
November 2, 2017 © 2017 The Authors. Published by Elsevier Inc.
https://doi.org/10.1016/j.molcel.2017.10.009

CellPress

Smc3 Deacetylation by Hos1 Facilitates Efficient Dissolution of Sister Chromatid Cohesion during Early Anaphase

Shuyu Li,[1] Zuojun Yue,[1] and Tomoyuki U. Tanaka[1,2,*]
[1]Centre for Gene Regulation and Expression, School of Life Sciences, University of Dundee, Dundee DD1 5EH, UK
[2]Lead Contact
*Correspondence: t.tanaka@dundee.ac.uk
https://doi.org/10.1016/j.molcel.2017.10.009

SUMMARY

Cohesins establish sister chromatid cohesion during S phase and are removed when cohesin Scc1 is cleaved by separase at anaphase onset. During this process, cohesin Smc3 undergoes a cycle of acetylation: Smc3 acetylation by Eco1 in S phase stabilizes cohesin association with chromosomes, and its deacetylation by Hos1 in anaphase allows re-use of Smc3 in the next cell cycle. Here we find that Smc3 deacetylation by Hos1 has a more immediate effect in the early anaphase of budding yeast. Hos1 depletion significantly delayed sister chromatid separation and segregation. Smc3 deacetylation facilitated removal of cohesins from chromosomes without changing Scc1 cleavage efficiency, promoting dissolution of cohesion. This action is probably due to disengagement of Smc1-Smc3 heads prompted by de-repression of their ATPase activity. We suggest Scc1 cleavage per se is insufficient for efficient dissolution of cohesion in early anaphase; subsequent Smc3 deacetylation, triggered by Scc1 cleavage, is also required.

INTRODUCTION

Cohesion between sister chromatids is established during DNA replication and removed when cells enter anaphase. Timely regulation of cohesion is crucial; if cohesion is lost precociously, or its removal is delayed, chromosome mis-segregation could result. Sister chromatid cohesion relies on the tetrameric cohesin complex, which is composed of Scc1 (also called Mcd1 or Rad21), Scc3, Smc1, and Smc3, which forms a ring structure embracing sister chromatids (Nasmyth and Haering, 2009).

Cohesins are loaded onto chromosomes in telophase or G1 phase, by the cohesin loader. During the subsequent S phase, Smc3 is acetylated by Eco1 acetyl-transferase (also called Ctf7). Smc3 acetylation prevents ATPase activity of the Smc1-Smc3 heads, which in turn inhibits opening of the Smc3-Scc1 interface (Chan et al., 2012; Beckouët et al., 2016; Çamdere et al., 2015; Elbatsh et al., 2016; Huber et al., 2016; Murayama and Uhlmann, 2015) and counteracts the activity of Wpl1 (also called Wapl and Rad61) (Rolef Ben-Shahar et al., 2008; Rowland et al., 2009; Unal et al., 2008; Zhang et al., 2008). Smc3 acetylation leads to replicated sister chromatids being stably trapped inside the cohesin ring complex. Sister chromatid cohesion is thus maintained until the onset of anaphase, when the cysteine protease separase becomes active and cleaves Scc1 (Nasmyth and Haering, 2009; Uhlmann, 2001). This leads to opening of the cohesin ring complex and removal of cohesion, allowing chromosome segregation to opposite spindle poles. During this process, Scc1 cleavage also allows Hos1 deacetylase to remove acetyl groups from Smc3 (Beckouët et al., 2010; Borges et al., 2010; Chan et al., 2013; Deardorff et al., 2012; Xiong et al., 2010). It is suggested that this deacetylation of Smc3 allows it to be re-used for cohesion in the next cell cycle, although this recycling is not essential for cell growth as new (and therefore non-acetylated) Smc3 is also expressed. These processes are essentially conserved from yeast to humans (Uhlmann, 2016).

It is unclear, however, whether Smc3 deacetylation has any immediate effect in regulating cohesins at anaphase onset when the deacetylation actually occurs. Moreover, although Scc1 cleavage leads to removal of cohesins from chromosomes (Uhlmann et al., 2000), it is unknown whether Scc1 cleavage itself is sufficient for this process or whether any events downstream of Scc1 cleavage are also involved. Here we address these questions using budding yeast as a model organism.

RESULTS

Cohesin Deacetylase Hos1 Promotes Efficient and High-Fidelity Sister Chromatid Segregation during Anaphase

To address the role of Smc3 deacetylation in anaphase, we aimed to abrogate the function of Hos1 deacetylase. Deletion of the *hos1* gene weakens sister chromatid cohesion, presumably due to a lack of Smc3 recycling for the next cell cycle (Beckouët et al., 2010; Borges et al., 2010). Therefore, to investigate immediate effects of abrogated Hos1 function in anaphase, we tagged the *HOS1* gene with an auxin-inducible degron and induced degradation by adding auxin (Nishimura et al., 2009). Hos1 was depleted within 20–40 min after adding auxin (Figure 1A). Smc3 deacetylation, which normally occurs in anaphase, was impaired after Hos1 depletion (Figure 1B), as reported in *hos1*-deleted cells (Beckouët et al., 2010; Borges et al., 2010; Xiong et al., 2010). After depletion of Hos1 following

CrossMark

This is an open access article under the CC BY license (http://creativecommons.org/licenses/by/4.0/).

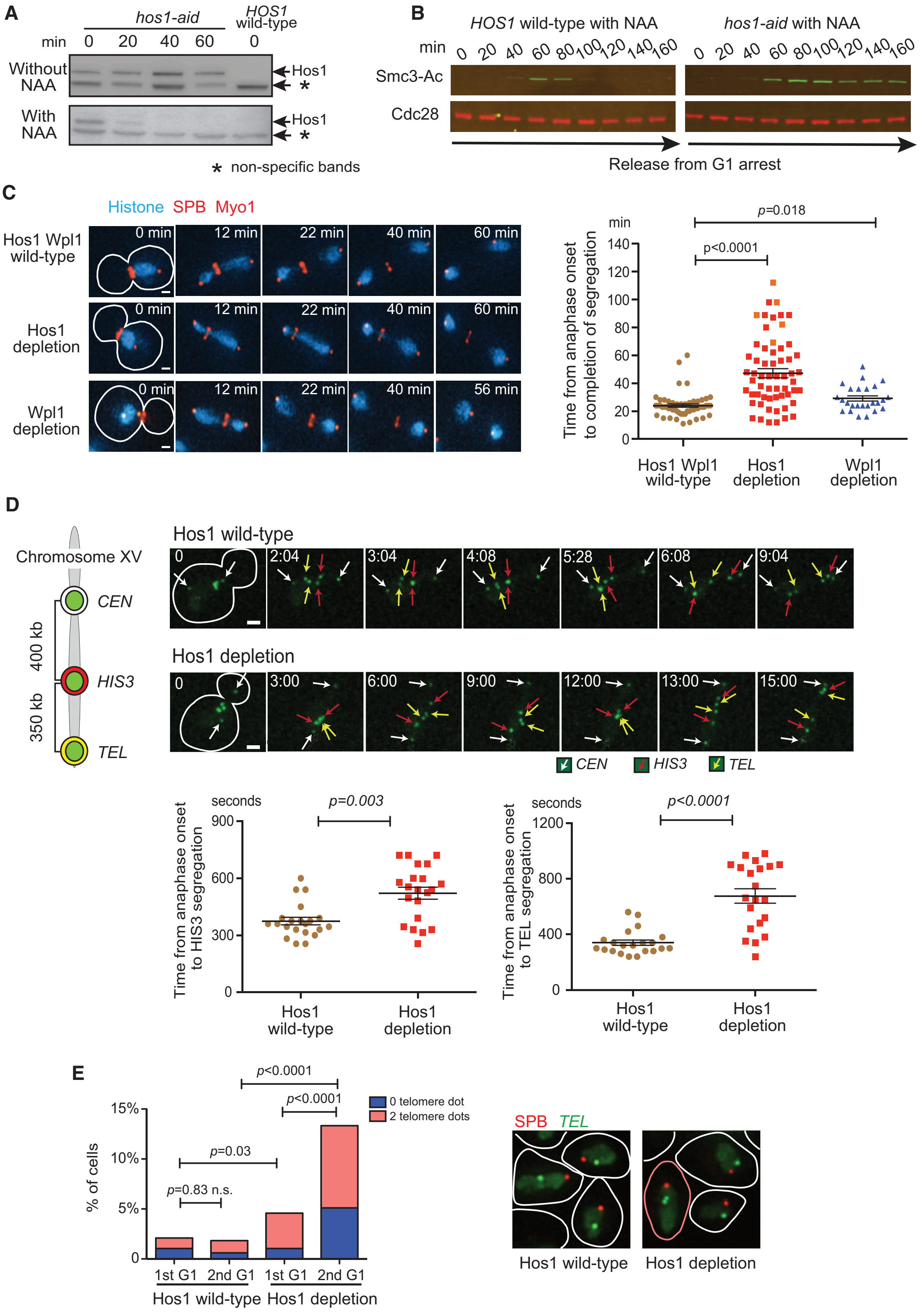

Figure 1. Depletion of Hos1 Leads to a Delay in Chromosome Segregation

(A) Hos1 was rapidly depleted using the auxin-induced degron system. Cells with (T11218) and without (T11219) an auxin-induced degron tag (*aid*) for *HOS1* were incubated with auxin (NAA 0.5 mM), and analyzed after 20, 40, and 60 min by western blotting with an anti-AID tag antibody.

(legend continued on next page)

G1 phase, sister chromatid cohesion was still maintained in metaphase as robustly as it was in Hos1 wild-type cells (Figure S1A).

After Hos1 depletion following G1 phase, we investigated chromosome segregation by visualizing chromosomes using histones fused with cyan fluorescent protein (CFP). In the same cells, we also visualized spindle poles with mCherry red fluorescent protein to evaluate spindle length and a myosin ring at the bud neck to monitor cytokinesis. In most of the wild-type cells, chromosomes completed segregation to opposite poles (i.e., histone signals split into two) within 30 min after anaphase onset, whereas in the majority of Hos1-depleted cells, it took substantially longer for chromosomes to complete segregation (Figure 1C). We also depleted Hos1 during metaphase arrest (rather than following G1), subsequently released cells to anaphase, and found similar outcomes (Figure S1B). We next analyzed segregation of a chosen chromosome (chromosome XV) by marking three loci (*CEN*, *HIS*, and *TEL*) with *tet* operators that could bind TetR-GFP (green fluorescent protein) fusion proteins, thus visualizing them as small GFP dots. In wild-type cells, *CEN*, *HIS*, and *TEL* dots—in that order—showed segregation to opposite poles (Renshaw et al., 2010). After Hos1 depletion following G1 phase, it took longer for these dots to complete their segregation (Figure 1D). Thus, chromosome segregation is substantially delayed during anaphase after Hos1 depletion.

In wild-type cells, anaphase spindle elongation takes place in two phases: an initial, rapid elongation and a subsequent, slower elongation (Straight et al., 1997). In Hos1-depleted cells, the initial, rapid spindle elongation occurred normally but the subsequent slower elongation was retarded and the completion of cytokinesis was significantly delayed (Figures S1C and S1D). We next addressed whether the spindle assembly checkpoint (SAC) is involved in the delay of chromosome segregation in Hos1-depleted cells. This was not the case, since Hos1-depleted cells showed a similar delay in chromosome segregation in the absence of the SAC (*mad2* deletion) (Figure S1E). We then analyzed the fidelity of chromosome segregation with Hos1 depletion. We visualized *TEL* (right telomere) on chromosome XV as a GFP dot. Following anaphase and cytokinesis with Hos1 depletion, the proportion of cells with no *TEL* dot or two *TEL* dots increased from 4.6% to 13.3% (Figure 1E). By contrast, such an increase was not found in Hos1 wild-type cells. Thus Hos1 depletion leads to an increased rate of chromosome mis-segregation.

Smc3 acetylation counteracts the Wpl1 function of opening the Smc3-Scc1 interface (see references in Introduction), so Hos1 depletion might delay chromosome segregation by counteracting the Wpl1 function. If so, we expected that Wpl1 depletion would show a similar outcome to that of Hos1 depletion. To test this, we depleted Wpl1 using an auxin-inducible degron (Figure S1F). After Wpl1 depletion, there was no substantial delay in chromosome segregation, in contrast to Hos1 depletion (Figure 1C). Collectively, Hos1 depletion delays chromosome segregation and increases chromosome mis-segregation. The delay in chromosome segregation is not dependent on the SAC or due to suppression of Wpl1 function.

Hos1 Facilitates Removal of Sister Chromatid Cohesion and Chromosome-Bound Cohesins during Early Anaphase without Affecting Scc1 Cleavage

Is the delay in chromosome segregation found with Hos1 depletion due to impaired regulation of sister chromatid cohesion? To address this, we visualized *CEN* and *HIS3* loci as GFP dots on chromosome XV (Figure 2A, diagram); *CEN* and *HIS3* dots were distinguishable, as the former segregated earlier and showed a higher intensity than the latter (see Figure 2B, cell images). We evaluated separation timing of sister *HIS3* loci after anaphase onset (Figure 2A, graph). In Hos1-depleted cells, sister

(B) Smc3 acetylation remained in anaphase after depletion of Hos1. Cells of T11219 and T11218 (see A) were released from G1 arrest to YPAD medium (at 0 min) containing NAA to deplete Hos1 aid. Mating pheromone was re-added to the culture after bud formation to re-arrest cells in the following G1 phase. The Smc3 acetylation (Smc3-Ac) was checked every 20 min by western blotting with anti-acetyl Smc3 (Borges et al., 2010) (top) and anti-Cdc28 (bottom, loading control) antibody.

(C) Segregation of chromosomes was delayed significantly after Hos1 depletion but only marginally after Wpl1 depletion. *hos1-aid* (T11218) and *wpl1-aid* (T11432) cells with *HTB2-CFP*, *SPC42-mCherry*, and *MYO1-mCherry*—where Htb2 is a histone H2B, Spc42 is a spindle-pole-body (SPB) component, and Myo1 is a component of the acto-myosin ring at the bud neck—were released from G1 arrest to YPAD medium containing 0.5 mM NAA. Control wild-type cells (T11219) were treated in the same way. From 80 min after release from G1, images were acquired every 2 min for 2 hr. Representative time-lapse images show chromosome segregation during anaphase (left; scale bar, 1 μm). Anaphase onset (time 0 in the image) is defined as the time when the distance between two SPBs reached 2.5 μm. The time of completion of chromosome segregation—i.e., disappearance of histone signals at the bud neck following their segregation—was plotted in individual cells (right). The bars in the graph show mean and SEM. Orange squares in middle (Hos1 depletion) show the time when time-lapse observation finished before chromosome segregation was completed. *p* values were obtained by t test. In the examples (left), chromosome segregation completed at 22, 44, and 22 min (from top to bottom). See Movies S1, S2, and S3.

(D) Segregation of *HIS3* and *TEL* loci on chromosome XV was delayed in Hos1-depleted cells. *HOS1* wild-type (T10830) and *hos1-aid* (T10829) cells with Tet repressors fused with GFP (*TetR-GFP*) and *tet* operators (*tetOs*) inserted at three loci on chromosome XV (*CEN*, *HIS3*, *TEL*; top left diagram) were treated as in (C). From 80 min after release from G1, images were acquired every 4 s for 45 min. Representative time-lapse images (top right) show segregation of the three GFP-labeled loci during anaphase (scale bar, 1 μm). The white, red, and yellow arrows indicate sister *CEN*, *HIS3*, and *TEL* loci, respectively, which were identified as they segregate in this order (Renshaw et al., 2010). Anaphase onset (time 0 in the image) was defined as the time when the distance between sister *CENs* reached 3 μm. Time required for *HIS3* and *TEL* locus segregation (defined as the time when *CEN–HIS* or *CEN–TEL* distance becomes < 1.5 μm in the bud) is plotted in the graph (bottom) where bars indicate mean and SEM. *p* values were obtained by t test. See Movies S4 and S5.

(E) Hos1 depletion leads to an increase in chromosome mis-segregation. *HOS1* wild-type (T11113) and *hos1-aid* (T11112) cells with *SPC42-mCherry*, *TetR-GFP*, and *TEL-tetOs* (on chromosome XV) were treated as in (B). Images were acquired during arrest in G1 (before NAA addition; 1st G1) and at 180 min after release from G1 arrest (after NAA treatment and cytokinesis; 2nd G1). Cells with zero and two *TEL* dots were counted (graph on left) and representative images are shown (right). The cell, highlighted in color, carried two *TEL* dots. Sample numbers (from left to right): n = 424, 657, 480, and 450. *p* values were obtained by Fisher's exact test (one versus zero/two dots). n.s., not significant.

See also Figure S1.

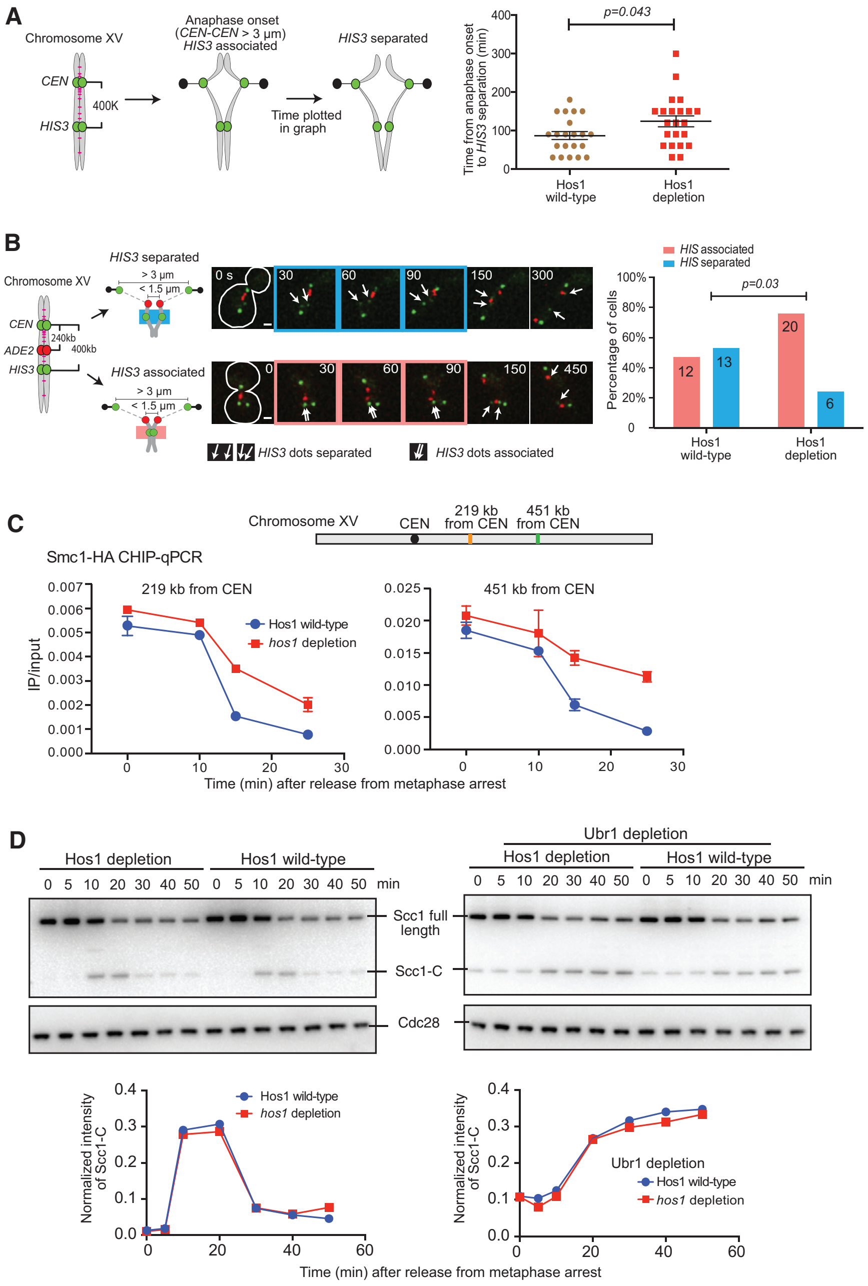

Figure 2. Hos1 Promotes Dissolution of Sister Chromatid Cohesion and Removal of Cohesins, without Affecting Scc1 Cleavage, during Early Anaphase

(A and B) Separation of sister *HIS3* loci after anaphase onset is delayed in Hos1-depleted cells. *HOS1* wild-type (T11710) and *hos1-aid* (T11713) cells with *TetR-GFP*, *3xCFP-LacI* (Lac repressor fused with three tandem copies of CFP), *tetOs* at *CEN* and *HIS3* loci, and *lacOs* at *ADE2* locus were treated as in Figure 1C. Anaphase onset was defined as in Figure 1D. In (A), images of GFP-labeled loci (*CEN*, *HIS3*) were collected every 30 s for 45 min from 80 min after release from G1 arrest (A, diagram). The timing of sister *HIS3* separation in individual cells was plotted (A, graph). The bars in the graph show mean and SEM. In (B), images of GFP- and CFP-labeled loci (*CEN*, *ADE2*, and *HIS3*) were collected. Representative time-lapse images in (B) show separation and segregation of *CEN*, *ADE2*, and *HIS3* loci (scale bar, 1 μm). We scored separation and non-separation of sister *HIS3* after anaphase onset but before the spindle force is applied (sister *ADE2* distance < 1.5 μm) (B, graph; sample numbers on bars). *p* values were obtained by t test (A) and Fisher's exact test (B).

(C) Removal of cohesins is delayed during early anaphase in Hos1-depleted cells. *HOS1* wild-type (T11875) and *hos1-aid* (T11874) cells with *SMC1-HA*, *SPC42-mCherry*, *MYO1-mCherry*, P_{GAL}-*CDC20* (*CDC20* expressed from *GAL1-10* promoter), and *cdc15-as* were released from G1 arrest to YPA medium with raffinose (to deplete Cdc20 for metaphase arrest). After 2.5 hr, galactose was added (defined as time 0) to release cells from metaphase arrest (by re-expressing *CDC20*). NAA and 1NM-PP1 were added for the last 1 hr during metaphase arrest, and after release from the arrest, to deplete Hos1-aid and to prevent completion of cytokinesis (by inhibiting Cdc15-as kinase), respectively. Cells were harvested at 10, 15, and 25 min and analyzed for Smc1-HA association at 219 and 451 kb from *CEN15* on chromosome XV (cohesin enrichment sites; Natsume et al., 2013) using ChIP-qPCR. The IP:input ratio in CHIP is plotted in graphs, where error bars show SD (n = 3).

(D) The efficiency of Scc1 cleavage was not affected by Hos1 depletion. *HOS1* wild-type (T11914), *hos1-aid* (T11912), *HOS1* wild-type *ubr1-aid* (T12726), and *hos1-aid ubr1-aid* (T12724) cells with *SCC1-HA*, *SPC42-mCherry*, *MYO1-mCherry*, P_{GAL}-*CDC20*, and *cdc15-as* were treated as in (C). Time 0 is defined as in (C). Samples were taken at indicated time points and analyzed by western blotting using anti-HA (top) and anti-Cdc28 (bottom, loading control) antibody. The amount of the C-terminal Scc1 cleavage product (Scc1-C) was quantified, normalized to that of Cdc28, and plotted in graphs.

See also Figure S2.

HIS3 dots took longer to separate, on average, than they did in wild-type cells. Sister *HIS3* separation may occur either before or after spindle pulling forces are applied to this locus through *CEN* and chromosome arms. We next evaluated the frequency of sister *HIS3* separation after anaphase onset but before any spindle-pulling force was applied on this locus. To monitor this, the *ADE2* locus was visualized as a CFP dot on chromosome XV (Figure 2B, diagram; Renshaw et al., 2010). We focused on the period when sister *CEN* dots were pulled toward opposite spindle poles but sister *ADE2* dots were not, and we evaluated separation of sister *HIS3* dots (Figure 2B). After Hos1 depletion, more cells showed associated (non-separated) sister *HIS3* dots during this period than was the case in wild-type cells (Figure 2B, graph). Thus, in Hos1-depleted cells, sister chromatid cohesion

persists longer in early anaphase before any spindle force is applied.

We next compared cellular defects caused by Hos1 depletion and *hos1* gene deletion (*hos1Δ*). Hos1 depletion freshly removed Hos1 in the current cell cycle, whereas in *hos1Δ* cells Hos1 was not functional in both previous and current cell cycles. In *hos1Δ* cells, cohesion was weakened in metaphase (Figure S2A, left), presumably due to failure in re-cycling Smc3 for the next cell cycle (Beckouët et al., 2010; Borges et al., 2010). Intriguingly, *hos1Δ* cells showed only a marginal delay in completing chromosome segregation, in contrast to cells with "fresh" Hos1 depletion (Figure S2A, right); probably a weaker cohesion in metaphase offset a delay in cohesion removal in anaphase, with *hos1Δ*.

Is the persistent cohesion found during early anaphase in Hos1-depleted cells due to a delay in the removal of cohesins? To address this question, we let cells synchronously enter anaphase (by Cdc20 depletion and subsequent re-expression) but prevented them from entering the next cell cycle (Figure S2B). In this situation, Hos1 wild-type and Hos1-depleted cells showed similar spindle elongation during anaphase (Figures S2C and S2D). We then quantified the amount of chromosome-bound cohesin Smc1 by chromatin immunoprecipitation and quantitative PCR (ChIP–qPCR) at two chromosome loci (Figure 2C). From 10 to 25 min after Cdc20 re-expression, most of the Smc1 was removed from chromosomes in wild-type cells, but removal was slower in Hos1-depleted cells. We also analyzed Smc1 enrichment using ChIP and high-throughput DNA sequencing (ChIP-seq) (Figure S2E). At 15 min after Cdc20 re-expression, peaks of Smc1 were still found above the background all along chromosomes in Hos1-depleted cells, whereas most of these were abolished in Hos1 wild-type cells. Thus, Hos1 depletion delays cohesin removal from chromosomes in early anaphase.

Scc1 cleavage by separase leads to removal of cohesins at the onset of anaphase (Uhlmann et al., 2000). We next addressed whether the efficiency of Scc1 cleavage was affected by Hos1 depletion. For this, we quantified the C-terminal Scc1 cleavage product (Scc1-C) upon entry into anaphase (Figure 2D, left). Scc1-C was detected at 10 and 20 min, and its amount was similar in wild-type and Hos1-depleted cells. Scc1-C degradation relies on Ubr1, which is the E3 ubiquitin ligase of the N-end rule pathway (Rao et al., 2001), and so we also quantified Scc1-C after Ubr1 depletion (Figure 2D, right). In this condition, the amount of Scc1-C increased at 20 min and subsequently remained high; the amount was similar in wild-type and Hos1-depleted cells. Thus, Hos1 depletion does not change the efficiency of Scc1 cleavage by separase. Hos1 facilitates removal of cohesins from chromosomes in early anaphase without changing Scc1 cleavage efficiency.

Smc3 Deacetylation by Hos1 at K112 and K113 Leads to Efficient Cohesin Removal in Anaphase and Timely Chromosome Segregation

We next addressed whether deacetylation of Smc3 by Hos1 is crucial for removal of cohesins from chromosomes and timely sister chromatid segregation. Smc3 is acetylated at lysines (K) 112 and 113 by Eco1 during S phase (Rolef Ben-Shahar et al., 2008; Unal et al., 2008) and deacetylated by Hos1 in early anaphase (Beckouët et al., 2010; Borges et al., 2010; Xiong et al., 2010). If Smc3 deacetylation by Hos1 at K112 and K113 is important for removal of cohesins in early anaphase, we can make the following two predictions: first, acetyl-Smc3 would be bound to anaphase chromosomes longer in Hos1-depleted cells; second, non-acetyl *smc3* mutants–e.g., replacement of lysines with arginines at these sites (K112R K113R)–would rescue timely chromosome segregation in Hos1-depleted cells.

We tested the first prediction by quantifying acetyl-Smc3 (at K112 and K113) that was bound to chromosomes. Acetyl-Smc3 was detected on fixed and immobilized chromosomes (Figure S3A). In metaphase, acetyl-Smc3 was detected at a similar level in Hos1 wild-type and Hos1-depleted cells (Figure 3A). However, in anaphase, more acetyl-Smc3 was detected on chromosomes in Hos1-depleted cells. For comparison, we quantified HA-tagged Smc3 on chromosomes, and this showed a similar behavior to that of acetyl-Smc3 (Figure S3B). Thus, acetyl-Smc3 indeed remains longer on anaphase chromosomes in Hos1-depleted cells.

It is not straightforward to test the second prediction, since non-acetyl *smc3* mutants (K112R K113R) are lethal (Rolef Ben-Shahar et al., 2008; Unal et al., 2008). This lethality is due to the destabilized Smc3-Scc1 interface, which hampers establishment of cohesion (Chan et al., 2012). We attempted to keep non-acetyl *smc3* mutant cells alive by fusing the C terminus of Smc3 to the N terminus of Scc1 (Smc3-Scc1). Cells with an Smc3-Scc1 fusion protein remain viable even if their original *SCC1* and *SMC3* are deleted (Gruber et al., 2006). We introduced non-acetyl K112R K113R mutations to the Smc3-Scc1 fusion and found that this non-acetyl Smc3-Scc1 fusion can maintain cell viability, as can the "wild-type" Smc3-Scc1 fusion (Figure S3C). The non-acetyl and "wild-type" Smc3-Scc1 fusions could establish and maintain cohesion similarly in metaphase, though cohesion in these cells is slightly weaker than in *SMC3+ SCC1+* (non-fusion) control cells (Figure S3D).

We then measured the period from anaphase onset to completion of chromosome segregation in these cells (Figure 3B). First, we found that in the presence of the Smc3-Scc1 fusion, Hos1 depletion caused a delay in completion of chromosome segregation (Figure 3B; left versus middle in graph). The delay by Hos1 depletion was smaller with the Smc3-Scc1 fusion than it was with non-fusion *SMC3+ SCC1+* (compare Figures 3B and 1C), presumably due to slightly weaker cohesion in metaphase (Figure S3D). Second, in Hos1-depleted cells, non-acetyl Smc3-Scc1 fusion alleviated a delay in chromosome segregation observed with"wild-type" Smc3-Scc1 fusion (Figure 3B; middle versus right in graph). Therefore, at least in the context of Smc3-Scc1 fusion, Smc3 non-acetyl mutants rescue timely chromosome segregation in Hos1-depleted cells. We also found that this rescue is independent of the SAC (Figure S3E). In summary, Smc3 K112 and K113 are Hos1 substrates whose deacetylation is crucial for efficient chromosome segregation.

Engineered Smc3 Cleavage by Separase Rescues Efficient Chromosome Segregation in Hos1-Depleted Cells

How does Smc3 deacetylation by Hos1 facilitate cohesin removal in early anaphase? It was suggested that Smc3

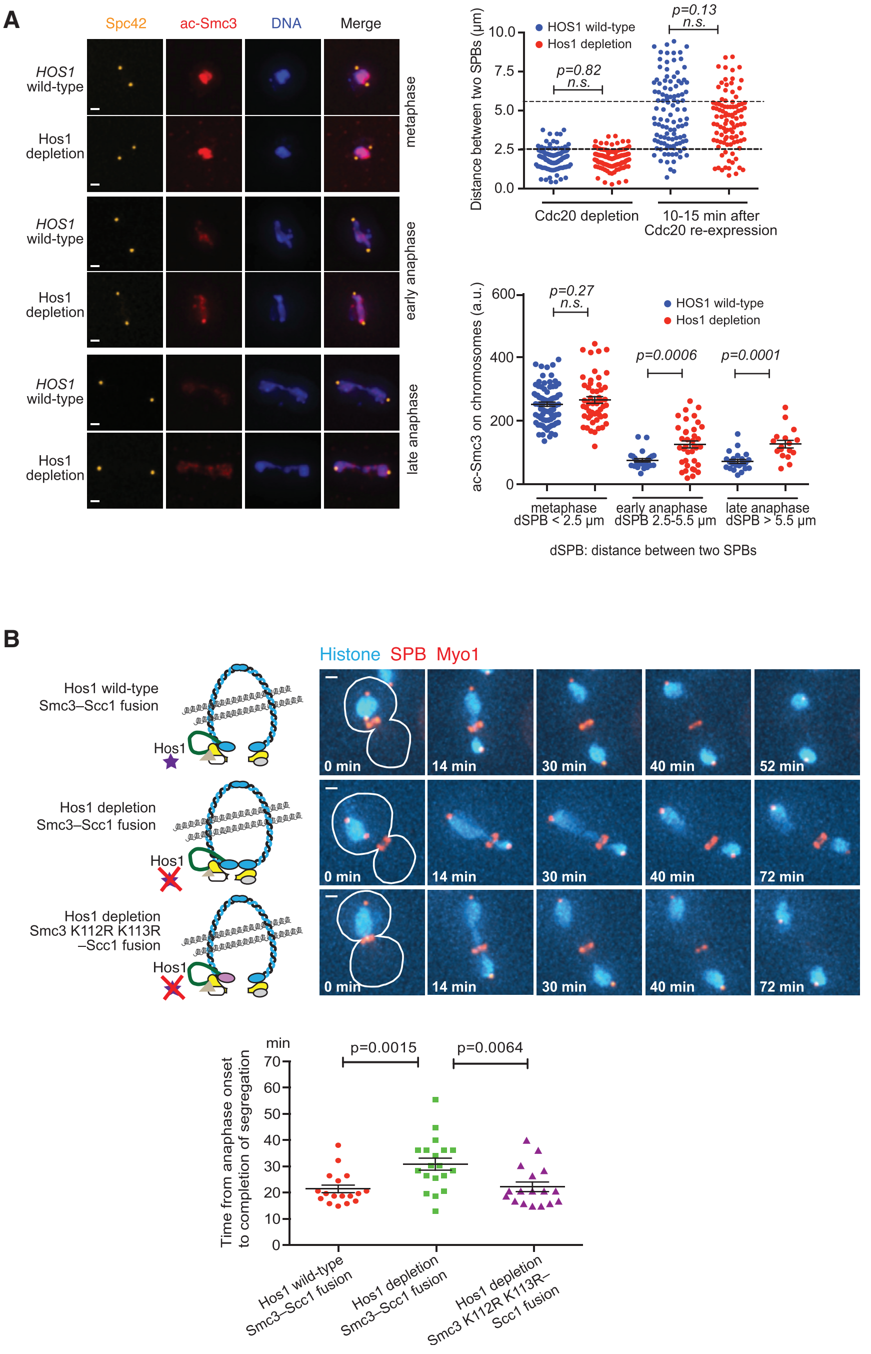

Figure 3. Smc3 Deacetylation at K112 and K113 by Hos1 Leads to Efficient Cohesin Removal in Anaphase and Timely Chromosome Segregation

(A) A larger amount of acetyl-Smc3 remains on anaphase chromosomes in Hos1-depleted cells. *HOS1* wild-type (T13179) and *hos1-aid* (T13180) cells with P_{GAL}-*CDC20 Spc42-mCherry* were arrested in metaphase and subsequently released to anaphase, as in Figure 2C. NAA was added to deplete Hos1-aid, as in Figure 2C. Cells in metaphase arrest and in anaphase (10–15 min after *CDC20* re-expression) were taken, and chromosomes were fixed and immobilized on a slide glass immediately after cell lysis. Representative cells are shown on the left. The distance between two SPBs (right, top) and acetyl-Smc3 (ac-Smc3) signals on chromosomes (right, bottom) were quantified. DNA signals showed elongation, and the distance between two SPBs was enlarged in this assay when cells proceeded from metaphase to anaphase (Renshaw et al., 2010). Cells in metaphase, early anaphase, and late anaphase were defined by SPB-SPB distance (right, bottom). Scale bars, 1 μm; a.u., arbitrary unit; *n.s.*, no significant difference. *p* value was obtained by t test. Bars and error bars show means and SEMs.

(B) Smc3 non-acetyl mutants rescue timely chromosome segregation in Hos1-depleted cells. *HOS1 wild-type SMC3-SCC1* fusion (T12684), *hos1-aid SMC3-SCC1* fusion (T12665), and *hos1-aid SMC3-K112R K113R–SCC1* fusion (T12666) cells with *SPC42-mCherry*, *MYO1-mCherry*, and *HTB2-CFP* were treated, and images were acquired as in Figure 1C. In these cells, the original *SMC3* and *SCC1* genes were deleted. Representative time-lapse images show chromosomes segregation during anaphase (scale bar, 1 μm). Anaphase onset (0 min) is defined, and time required for completion of chromosome segregation was analyzed (graph) as in Figure 1C. *p* values were obtained by t test. In the examples shown, chromosome segregation completed at 24, 42, and 24 min (from top to bottom).

See also Figure S3.

acetylation inhibits ATPase activity of Smc1-Smc3 heads and that, while ATP hydrolysis is prohibited, Smc1-Smc3 heads remain engaged (Beckouët et al., 2016; Çamdere et al., 2015; Elbatsh et al., 2016; Huber et al., 2016; Murayama and Uhlmann, 2015) (Figure 4A). Smc3 deacetylation could disengage Smc1-Smc3 heads and thus facilitate the exit of chromosomes from the ring composed of Smc1 and Smc3 (and from the entire cohesin ring, since Scc1 is cleaved at anaphase onset). This model would give the following prediction: if there were a way to open up the Smc1-Smc3 ring without relying on opening Smc1-Smc3 heads, it would expedite cohesin removal in Hos1-depleted cells. One way to do so is to cut Smc3 (or Smc1) in its coiled-coil region. For this, we inserted separase cleavage sites into the Smc3 coiled-coil region (Figure 4B, diagram, Smc3-2R). These insertion sites were selected so as not to perturb the Smc3 coiled-coil structure (Gruber et al., 2003). As a control, mutated separase cleavage sites were also inserted in the same way (Smc3-2D).

When cells expressed Smc3-2R or -2D as the sole Smc3 protein, these Smc3 mutants supported robust cohesion as did Smc3 wild-type (Figure S4A). As expected, we could detect an Smc3 cleavage product with Smc3-2R, but not with Smc3-2D (Figure 4B, lanes 2 and 3). To confirm that the engineered Smc3 cleavage is able to open the Smc1-Smc3 ring, we asked

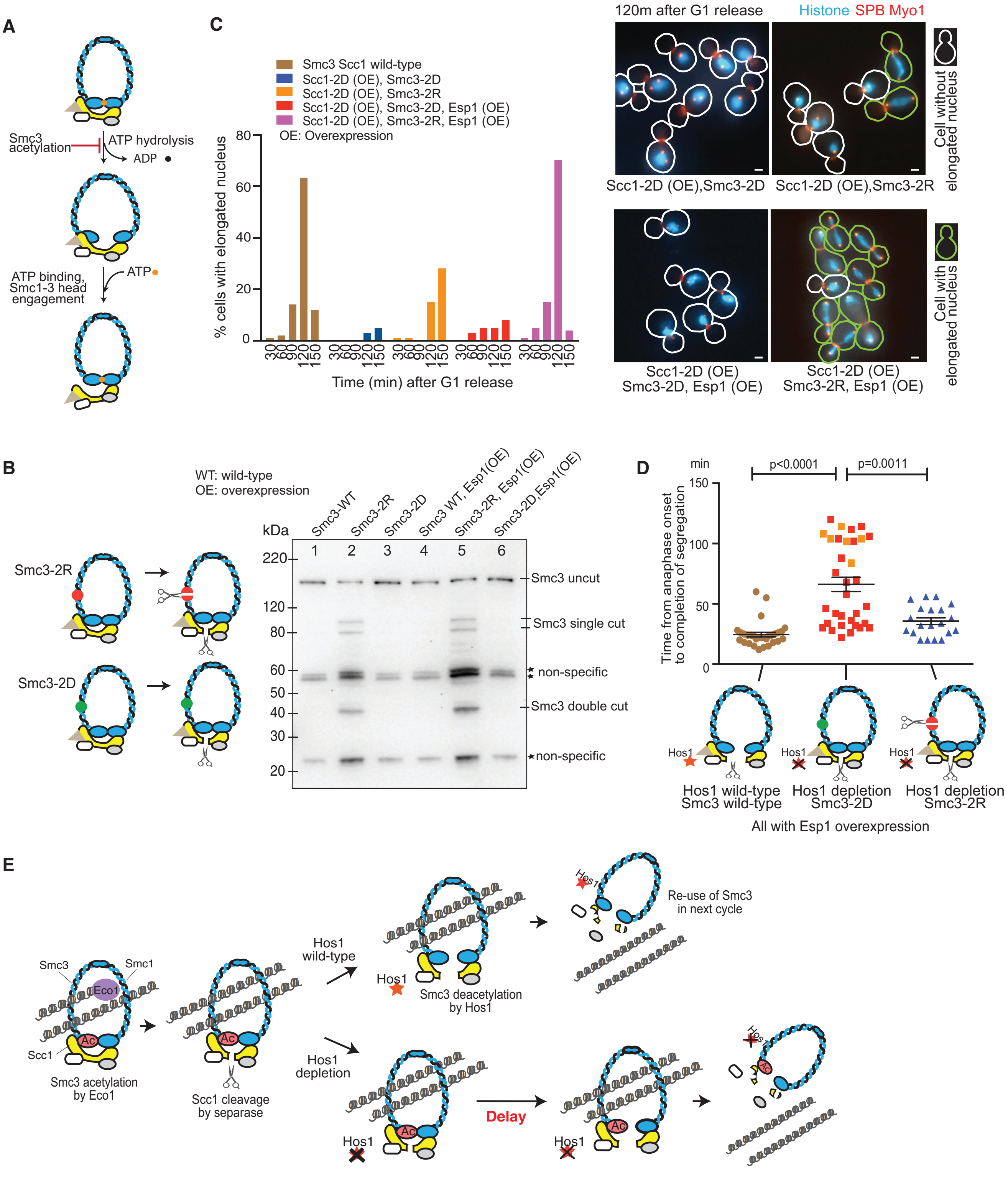

Figure 4. Engineered Smc3 Cleavage by Separase Rescues Efficient Chromosome Segregation in Hos1-Depleted Cells

(A) Diagram shows that Smc3 acetylation inhibits ATPase activity of Smc1-Smc3 heads, and Smc1-Smc3 heads remain engaged until ATP is hydrolyzed (top) (Beckouët et al., 2016; Çamdere et al., 2015; Elbatsh et al., 2016; Huber et al., 2016; Murayama and Uhlmann, 2015). In the absence of Smc3 acetylation, ATP is hydrolyzed and Smc1-Smc3 heads are disengaged (middle). Subsequent ATP binding leads to opening of the Smc3-Scc1 interface and destabilizes cohesin association with chromosomes (bottom).

(legend continued on next page)

if Smc3-2R could rescue chromosome segregation in the presence of an Scc1 whose separase cleavage sites were mutated (Scc1-2D; Uhlmann et al., 1999). Indeed, in the presence of Scc1-2D, we observed a partial rescue of chromosome segregation by Smc3-2R, but not by Smc3-2D (Figure 4C, orange and blue bars). Since Smc3 is not a natural target of separase, Smc3-2R cleavage by separase may not be efficient, but it may be made more so if separase is overexpressed. With separase overexpression (Figure S4B), cohesin regulation was largely normal (Figures S4C and S4D), but Smc3-2R showed a higher rate of chromosome segregation in the presence of Scc1-2D (Figure 4C, magenta bars). Thus, the engineered cleavage of Smc3 by separase can open the cohesin ring when non-cleavable Scc1 is present.

We then addressed whether the Smc3 cleavage (with separase overexpression) rescues timely chromosome segregation in Hos1-depleted cells (Figure 4D). Hos1 depletion delayed chromosome segregation in the majority of cells with Smc3-2D. Importantly, this delay was significantly alleviated with Smc3-2R in Hos1-depleted cells. By contrast, in Hos1 wild-type cells, neither Smc3-2D nor Smc3-2R changed the timing of chromosome segregation (Figure S4E). Thus, the engineered Smc3 cleavage can rescue timely chromosome segregation in Hos1-depleted cells. The result is consistent with Smc3 deacetylation by Hos1 facilitating disengagement of the Smc1-Smc3 heads to promote removal of cohesins from chromosomes at anaphase onset.

DISCUSSION

Our study suggests that Smc3 deacetylation by Hos1 facilitates cohesin removal and efficient dissolution of sister chromatid cohesion in early anaphase (Figure 4E). This action is presumably due to de-repression of the ATPase activity of Smc1-Smc3 heads prompted by Smc3 deacetylation, which leads to Smc1-Smc3 head disengagement. It has been shown that Smc3 deacetylation by Hos1 is trigged by Scc1 cleavage by separase (Beckouët et al., 2010; Chan et al., 2013). Thus Scc1 cleavage and subsequent Smc1-Smc3 head disengagement together open up the cohesin ring complex, facilitating sister chromatid separation and segregation. Both steps are required to fully open up the cohesin ring complex. In this context, Scc1 cleavage per se is not sufficient for efficient removal of cohesin; Smc3 deacetylation of Hos1 (which is trigged by Scc1 cleavage) is also required. It has been thought that Scc1 cleavage by separase is the final regulatory step to release cohesin and to dissolve cohesion at anaphase onset. Our results provide evidence that there is another regulatory step required for efficient cohesin release and cohesion dissolution on chromosomes. Nevertheless, while Scc1 cleavage is essential for cohesin removal (Uhlmann et al., 1999), Smc3 deacetylation by Hos1 is important only for efficient cohesin removal (Figure 2C). Without Hos1, Smc1-Smc3 head disengagement would be delayed but would eventually occur (Figure 4E). We assume that Smc1-Smc3 head disengagement takes place spontaneously, but with a delay, in Hos1-depleted cells.

Our results suggest that Smc3 K112 and K113 are important targets of Hos1, whose deacetylation promotes chromosome segregation in early anaphase (Figure 3). However, we cannot rule out that deacetylation of additional targets by Hos1 also contributes to this process. For example, it has been recently reported that coiled-coil regions of Smc1 and Smc3 are acetylated (in addition to Smc3 K112 and K113), facilitating association of Smc1 and Smc3 in these regions (Kulemzina et al., 2016). It is currently unknown whether Hos1 deacetylates these sites. If this is the case, it is possible that deacetylation of these sites by Hos1 facilitates dissociation of Smc1 and Smc3 in their coiled-coil regions and promotes removal of cohesins after Scc1 cleavage.

The cohesin structure and the Smc3 acetylation cycle are well conserved in evolution from yeast to humans. It is therefore

(B) Cleavage products are detected in cells carrying *SMC3* with separase cleavage sites (*SMC3-2R*) but not in control (*SMC3-2D*). Cells with *SMC3* wild-type (T12975), *SMC3-2R* (T11116), *SMC3-2D* (T11117), *SMC3* wild-type P_{GAL}-*ESP1* (T12976), *SMC3-2R* P_{GAL}-*ESP1* (T12892), and *SMC3-2D* P_{GAL}-*ESP1* (T12893) were grown in YPA medium containing raffinose. Expression of *ESP1* was induced by addition of galactose for 2.5 hr. In all cells, *SMC3* wild-type and mutants were tagged with *myc* epitopes at their C terminus, and the original untagged wild-type *SMC3* was deleted, while the original *ESP1* was intact. Smc3 was detected by western blotting with an anti-myc antibody.

(C) Engineered Smc3 cleavage by separase could rescue chromosome segregation in the presence of Scc1 non-cleavable mutant (*SCC1-2D*), and this rescue was more efficient when separase was overexpressed. *SMC3-2D* (T12201), *SMC3-2R* (T12200), *SMC3-2D* P_{GAL}-*ESP1* (T12827), and *SMC3-2R* P_{GAL}-*ESP1* (T12825) cells with P_{GAL}-*SCC1-2D, HTB2-CFP*, *SPC42-mCherry*, and *MYO1-mCherry* were released from G1 arrest to YPA medium with raffinose. Galactose was added for the last 30 min during G1 arrest and subsequent release from G1 to express *SCC1-2D* (and to overexpress *ESP1* in relevant strains). In all these cells, the original wild-type *SMC3* was deleted, while the original *ESP1* was intact. Control *SMC3 SCC1* wild-type cells (T11219) were treated in the same way. Elongated nuclei were defined as those with two SPBs apart > 3.0 μm; their percentage is shown in graph (left). In representative images at 120 min after release from G1 (right), cells with elongated nuclei are highlighted with green outlines.

(D) Engineered Smc3 cleavage by separase rescues timely chromosome segregation in Hos1-depleted cells. *HOS1 SMC3* wild-type (T12986), *hos1-aid SMC3-2D* (T12565), and *hos1-aid SMC3-2R* (T12566) cells with P_{GAL}-*ESP1*, *HTB2-CFP*, *Spc42-mCherry*, and *Myo1-mCherry* were treated, and images were acquired as in Figure 1C. Time from anaphase onset to completion of chromosome segregation was plotted as in Figure 1C. Orange squares are as in Figure 1C. *p* values were obtained by t test.

(E) Summary of the outcomes of Smc3 deacetylation by Hos1. Top: Scc1 cleavage by separase prompts deacetylation of Smc3 by Hos1 (Beckouët et al., 2010; Borges et al., 2010; Chan et al., 2013), which in turn facilitates disengagement of Smc1-Smc3 heads (Beckouët et al., 2016; Çamdere et al., 2015; Elbatsh et al., 2016; Huber et al., 2016; Murayama and Uhlmann, 2015), leading to efficient removal of cohesins from chromosomes and rapid dissolution of sister chromatid cohesion (current study). Deacetylated Smc3 is re-used in the next cell cycle (Beckouët et al., 2010; Borges et al., 2010; Xiong et al., 2010). Bottom: Without Hos1, Smc3 acetylation remains in anaphase after cleavage of Scc1 by separase, causing a delay in removal of cohesin from chromosomes and in dissolution of cohesion. Acetylated Smc3 cannot be used in the next cell cycle.

See also Figure S4.

likely that the mechanism found in yeast in this study is conserved in higher eukaryotes. Indeed, HDAC8 has been identified as the Smc3 deacetylase in human cells, and its inactivation leads to longer retention of cohesins, including cleaved Scc1, on chromosomes during anaphase (Deardorff et al., 2012). Further study may find a delay in chromosome segregation and cytokinesis with inactive HDAC8 in human cells, as we have found here in budding yeast. Mutations in HDAC8 are associated with Cornelia de Lange syndrome, a dominantly inherited congenital disorder (Deardorff et al., 2012). It is possible that delays in chromosome segregation are associated with development of the disease.

STAR★METHODS

Detailed methods are provided in the online version of this paper and include the following:

- KEY RESOURCES TABLE
- CONTACT FOR REAGENT AND RESOURCE SHARING
- EXPERIMENTAL MODEL AND SUBJECT DETAILS
- METHOD DETAILS
  - Yeast strains and cell culture
  - Construction of Smc3 cleavable by separase
  - Live-cell imaging and image analysis
  - Western blotting
  - Chromatin immunoprecipitation
  - Chromosome spreads (chromosome fixation and immobilization)
- QUANTIFICATION AND STATISTICAL ANALYSIS
- DATA AND SOFTWARE AVAILABILITY

SUPPLEMENTAL INFORMATION

Supplemental Information includes four figures, one table, and five movies and can be found with this article online at https://doi.org/10.1016/j.molcel.2017.10.009.

AUTHOR CONTRIBUTIONS

Conceptualization, Methodology, Visualization, and Writing (Original Draft) – S.L. and T.U.T.; Investigation, Formal Analysis, and Validation – S.L. and Z.Y.; Resources and Data Curation – S.L.; Funding Acquisition, Supervision, Project Administration, and Writing (Review & Editing) – T.U.T.

ACKNOWLEDGMENTS

We thank Tanaka lab members and K. Nasmyth for discussion; L. Clayton for reading the manuscript; M. Gierlinski for analyzing ChIP-seq data; S. Swift, G. Ball, P. Appleton, and L. Clayton for technical help; Edinburgh Genomics for high-throughput DNA sequencing; K. Shirahige, R. Ciosk, J.E. Haber, M. Kanemaki, K. Nasmyth, N. O'Reilly, K.E. Sawin, A. Straight, R.Y. Tsien, F. Uhlmann, EUROSCARF, and Yeast Resource Centre for reagents. This work was supported by an ERC advanced grant (322682), the Wellcome Trust (096535 and 097945) and the Medical Research Council (K015869). T.U.T. is a Wellcome Trust Principal Research Fellow.

Received: February 22, 2017
Revised: September 7, 2017
Accepted: October 6, 2017
Published: November 2, 2017

REFERENCES

Amberg, D.C., Burke, D.J., and Strathern, J.N. (2005). Methods in yeast genetics (CSHL Press).

Beckouët, F., Hu, B., Roig, M.B., Sutani, T., Komata, M., Uluocak, P., Katis, V.L., Shirahige, K., and Nasmyth, K. (2010). An Smc3 acetylation cycle is essential for establishment of sister chromatid cohesion. Mol. Cell *39*, 689–699.

Beckouët, F., Srinivasan, M., Roig, M.B., Chan, K.L., Scheinost, J.C., Batty, P., Hu, B., Petela, N., Gligoris, T., Smith, A.C., et al. (2016). Releasing activity disengages cohesin's Smc3/Scc1 interface in a process blocked by acetylation. Mol. Cell *61*, 563–574.

Borges, V., Lehane, C., Lopez-Serra, L., Flynn, H., Skehel, M., Rolef Ben-Shahar, T., and Uhlmann, F. (2010). Hos1 deacetylates Smc3 to close the cohesin acetylation cycle. Mol. Cell *39*, 677–688.

Çamdere, G., Guacci, V., Stricklin, J., and Koshland, D. (2015). The ATPases of cohesin interface with regulators to modulate cohesin-mediated DNA tethering. eLife *4*, e11315.

Chan, K.L., Roig, M.B., Hu, B., Beckouët, F., Metson, J., and Nasmyth, K. (2012). Cohesin's DNA exit gate is distinct from its entrance gate and is regulated by acetylation. Cell *150*, 961–974.

Chan, K.L., Gligoris, T., Upcher, W., Kato, Y., Shirahige, K., Nasmyth, K., and Beckouët, F. (2013). Pds5 promotes and protects cohesin acetylation. Proc. Natl. Acad. Sci. USA *110*, 13020–13025.

D'Aquino, K.E., Monje-Casas, F., Paulson, J., Reiser, V., Charles, G.M., Lai, L., Shokat, K.M., and Amon, A. (2005). The protein kinase Kin4 inhibits exit from mitosis in response to spindle position defects. Mol. Cell *19*, 223–234.

Deardorff, M.A., Bando, M., Nakato, R., Watrin, E., Itoh, T., Minamino, M., Saitoh, K., Komata, M., Katou, Y., Clark, D., et al. (2012). HDAC8 mutations in Cornelia de Lange syndrome affect the cohesin acetylation cycle. Nature *489*, 313–317.

Elbatsh, A.M.O., Haarhuis, J.H.I., Petela, N., Chapard, C., Fish, A., Celie, P.H., Stadnik, M., Ristic, D., Wyman, C., Medema, R.H., et al. (2016). Cohesin releases DNA through asymmetric ATPase-driven ring opening. Mol. Cell *61*, 575–588.

Feng, J., Liu, T., Qin, B., Zhang, Y., and Liu, X.S. (2012). Identifying ChIP-seq enrichment using MACS. Nat. Protoc. *7*, 1728–1740.

Gruber, S., Haering, C.H., and Nasmyth, K. (2003). Chromosomal cohesin forms a ring. Cell *112*, 765–777.

Gruber, S., Arumugam, P., Katou, Y., Kuglitsch, D., Helmhart, W., Shirahige, K., and Nasmyth, K. (2006). Evidence that loading of cohesin onto chromosomes involves opening of its SMC hinge. Cell *127*, 523–537.

Huber, R.G., Kulemzina, I., Ang, K., Chavda, A.P., Suranthran, S., Teh, J.T., Kenanov, D., Liu, G., Rancati, G., Szmyd, R., et al. (2016). Impairing Cohesin Smc1/3 Head Engagement Compensates for the Lack of Eco1 Function. Structure *24*, 1991–1999.

Kalantzaki, M., Kitamura, E., Zhang, T., Mino, A., Novák, B., and Tanaka, T.U. (2015). Kinetochore-microtubule error correction is driven by differentially regulated interaction modes. Nat. Cell Biol. *17*, 530.

Kubota, T., Nishimura, K., Kanemaki, M.T., and Donaldson, A.D. (2013). The Elg1 replication factor C-like complex functions in PCNA unloading during DNA replication. Mol. Cell *50*, 273–280.

Kulemzina, I., Ang, K., Zhao, X., Teh, J.T., Verma, V., Suranthran, S., Chavda, A.P., Huber, R.G., Eisenhaber, B., Eisenhaber, F., et al. (2016). A reversible association between Smc coiled coils is regulated by lysine acetylation and is required for cohesin association with the DNA. Mol. Cell *63*, 1044–1054.

Murayama, Y., and Uhlmann, F. (2015). DNA entry into and exit out of the cohesin ring by an interlocking gate mechanism. Cell *163*, 1628–1640.

Nasmyth, K., and Haering, C.H. (2009). Cohesin: its roles and mechanisms. Annu. Rev. Genet. *43*, 525–558.

Natsume, T., Müller, C.A., Katou, Y., Retkute, R., Gierliński, M., Araki, H., Blow, J.J., Shirahige, K., Nieduszynski, C.A., and Tanaka, T.U. (2013). Kinetochores

CellPress

coordinate pericentromeric cohesion and early DNA replication by Cdc7-Dbf4 kinase recruitment. Mol. Cell *50*, 661–674.

Nishimura, K., Fukagawa, T., Takisawa, H., Kakimoto, T., and Kanemaki, M. (2009). An auxin-based degron system for the rapid depletion of proteins in nonplant cells. Nat. Methods *6*, 917–922.

O'Reilly, N., Charbin, A., Lopez-Serra, L., and Uhlmann, F. (2012). Facile synthesis of budding yeast a-factor and its use to synchronize cells of α mating type. Yeast *29*, 233–240.

Rao, H., Uhlmann, F., Nasmyth, K., and Varshavsky, A. (2001). Degradation of a cohesin subunit by the N-end rule pathway is essential for chromosome stability. Nature *410*, 955–959.

Renshaw, M.J., Ward, J.J., Kanemaki, M., Natsume, K., Nédélec, F.J., and Tanaka, T.U. (2010). Condensins promote chromosome recoiling during early anaphase to complete sister chromatid separation. Dev. Cell *19*, 232–244.

Rolef Ben-Shahar, T., Heeger, S., Lehane, C., East, P., Flynn, H., Skehel, M., and Uhlmann, F. (2008). Eco1-dependent cohesin acetylation during establishment of sister chromatid cohesion. Science *321*, 563–566.

Rowland, B.D., Roig, M.B., Nishino, T., Kurze, A., Uluocak, P., Mishra, A., Beckouët, F., Underwood, P., Metson, J., Imre, R., et al. (2009). Building sister chromatid cohesion: smc3 acetylation counteracts an antiestablishment activity. Mol. Cell *33*, 763–774.

Straight, A.F., Marshall, W.F., Sedat, J.W., and Murray, A.W. (1997). Mitosis in living budding yeast: anaphase A but no metaphase plate. Science *277*, 574–578.

Sullivan, M., Hornig, N.C., Porstmann, T., and Uhlmann, F. (2004). Studies on substrate recognition by the budding yeast separase. J. Biol. Chem. *279*, 1191–1196.

Tanaka, T., Knapp, D., and Nasmyth, K. (1997). Loading of an Mcm protein onto DNA replication origins is regulated by Cdc6p and CDKs. Cell *90*, 649–660.

Tanaka, K., Kitamura, E., and Tanaka, T.U. (2010). Live-cell analysis of kinetochore-microtubule interaction in budding yeast. Methods *51*, 206–213.

Uhlmann, F. (2001). Secured cutting: controlling separase at the metaphase to anaphase transition. EMBO Rep. *2*, 487–492.

Uhlmann, F. (2016). SMC complexes: from DNA to chromosomes. Nat. Rev. Mol. Cell Biol. *17*, 399–412.

Uhlmann, F., Lottspeich, F., and Nasmyth, K. (1999). Sister-chromatid separation at anaphase onset is promoted by cleavage of the cohesin subunit Scc1. Nature *400*, 37–42.

Uhlmann, F., Wernic, D., Poupart, M.A., Koonin, E.V., and Nasmyth, K. (2000). Cleavage of cohesin by the CD clan protease separin triggers anaphase in yeast. Cell *103*, 375–386.

Unal, E., Heidinger-Pauli, J.M., Kim, W., Guacci, V., Onn, I., Gygi, S.P., and Koshland, D.E. (2008). A molecular determinant for the establishment of sister chromatid cohesion. Science *321*, 566–569.

Wloka, C., and Bi, E. (2012). Mechanisms of cytokinesis in budding yeast. Cytoskeleton *69*, 710–726.

Xiong, B., Lu, S., and Gerton, J.L. (2010). Hos1 is a lysine deacetylase for the Smc3 subunit of cohesin. Curr. Biol. *20*, 1660–1665.

Zhang, J., Shi, X., Li, Y., Kim, B.J., Jia, J., Huang, Z., Yang, T., Fu, X., Jung, S.Y., Wang, Y., et al. (2008). Acetylation of Smc3 by Eco1 is required for S phase sister chromatid cohesion in both human and yeast. Mol. Cell *31*, 143–151.

STAR★METHODS

KEY RESOURCES TABLE

REAGENT or RESOURCE	SOURCE	IDENTIFIER
Antibodies		
Mouse anti acetyl-Smc3 antibody	Katsuhiko Shirahige lab (Borges et al., 2010)	N/A
Rabbit polyclonal anti-AID antibody	Masato Kanemaki lab	N/A
Mouse anti HA-tag antibody [16B12]	Covance Research Products Inc.	MMS-101R-200; RRID: AB_291263
Mouse anti myc-tag antibody [9E11]	Abcam	Ab56; RRID: AB_304976
Goat anti Cdc28 polyclonal IgG [yC20]	Santa Cruz	Sc-6709; RRID: AB_671808
Goat anti-mouse IgG secondary antibody (Alexa Fluor®647)	Abcam	Ab150119
Donkey anti-goat IgG secondary antibody (HRP)	Abcam	Ab6885; RRID: AB_955423
Sheep anti-mouse IgG secondary antibody (HRP)	GE Healthcare Life Sciences	NA931; RRID: AB_772210
Donkey anti-rabbit IgG secondary antibody (HRP)	GE Healthcare Life Sciences	NA934; RRID: AB_772206
Donkey anti-mouse IRDye®800CW	Li-COR Biosciences	926-32212; RRID: AB_621847
Donkey anti-Goat IRDye®680RD	Li-COR Biosciences	924-68074; RRID: AB_10956736
Bacterial and Virus Strains		
DH5α competent cells	Thermo Fisher Scientific	18265017
XL1-Blue competent cells	Agilent	200249
Chemicals, Peptides, and Recombinant Proteins		
PP1 analog II, 1NM-PP1	Calbiochem	221244-14-0
1-Naphthaleneacetic acid (NAA)	Sigma-Aldrich	N0640-25G
α factor	Pepceuticals Ltd	N/A
a factor	Nicola O'Reilly (O'Reilly et al., 2012)	N/A
Dynabeads® protein A	Thermo Fisher Scientific	10001D
Hoechst 33342	Thermo Fisher Scientific	62249
SYBR Green PCR kit	QIAGEN	204074
Concanavalin A	Sigma-Aldrich	C7275
Deposited Data		
CHIP-seq data	This paper	GEO: GSE96841
Original image files (Mendeley data)	This paper	https://doi.org/10.17632/m64mz2tbvh.1
Experimental Models: Organisms/Strains		
S. cerevisiae W303 and its derivatives (see Table S1 for detail)	This study	See Table S1
Oligonucleotides		
PCR primers for amplifying 219 kb region from *CEN15* (in Figure 2C) 5′-TGGGACGTATGATTGTTGAGG-3′ and 5′-GGAGCCATTAACGTGGTCAT-3′	This study	N/A
PCR primers for amplifying 451 kb region from *CEN15* (in Figure 2C) 5′-AAGAAGGAGCATGAGGGTTTGAG-3′ and 5′-CCAGGAACATGCAGTGCGTTAAG-3′	This study	N/A
Recombinant DNA		
SMC3-2D (*LEU2* marker)	This study	pT2882
SMC3-2R (*LEU2* marker)	This study	pT2883

(*Continued on next page*)

Continued

REAGENT or RESOURCE	SOURCE	IDENTIFIER
SMC3–SCC1 fusion (*URA3* marker)	Kim Nasmyth lab (Gruber et al., 2006)	K4879
SMC3 K112R K113R–SCC1 fusion (*URA3* marker)	This study	pT2941
P_{GAL}-*SCC1 R180D, R268D* (*ADE2* marker)	Kim Nasmyth lab (Uhlmann et al., 1999) and this study	pT2843
Software and Algorithms		
Volocity 6.2.1	PerkinElmer	http://www.perkinelmer.com/
Imaris 7.7.2	Bitplane	http://www.bitplane.com
Prism 6.0	GraphPad	https://www.graphpad.com/
ImageLab 4.1	Bio-Rad	http://www.bio-rad.com/en-ch/product/image-lab-software
MACS 2.1.0 (Model-based Analysis of CHIP-seq)	Shirley Liu lab (Feng et al., 2012)	http://liulab.dfci.harvard.edu/MACS/
Integrative Genomic Viewer 2.4	Broad Institute	https://software.broadinstitute.org/software/igv/download
SoftWoRX® 6.5.2	GE Healthcare	http://incelldownload.gehealthcare.com/bin/download_data/SoftWoRx/6.5.2/SoftWoRx.htm
Image Studio Lite3.1	Li-Cor	https://www.licor.com/bio/products/software/image_studio_lite/download.html
Other		
Glass-bottom dish	MatTek	P35G-1.5-10-C

CONTACT FOR REAGENT AND RESOURCE SHARING

Requests for further information or reagents should be directed to the Lead Contact, Tomoyuki U. Tanaka (t.tanaka@dundee.ac.uk).

EXPERIMENTAL MODEL AND SUBJECT DETAILS

All yeast *Saccharomyces cerevisiae* strains, used in this study, had a W303 background (derived from K699 and K700 from the Nasmyth laboratory). The genotypes of the yeast strains are shown in Table S1.

METHOD DETAILS

Yeast strains and cell culture

Methods for yeast culture have been described previously (Amberg et al., 2005; Renshaw et al., 2010). To synchronize cells in the cell cycle, yeast cells were arrested in G1 phase by treatment with yeast mating hormone (α or a factor) and subsequently released to fresh media (Amberg et al., 2005; O'Reilly et al., 2012). Cells were cultured at 25°C in YPA medium (1% yeast extract, 2% peptone, 0.01% adenine hydrochloride) containing 2% glucose (YPAD), unless otherwise stated. To activate *MET3* promoter, cells were incubated in methionine drop-out medium. The *MET3* promoter was suppressed by adding 2 mM methionine to the relevant medium. To activate *GAL1-10* promoter, cells were incubated in YPA medium containing 2% raffinose for at least 2.5 h, and subsequently 2% galactose was added. *SPC42*, *MYO1* and *NIC96* genes were tagged with mCherry or with 4×mCherry at their C terminus at their original gene loci using a one-step PCR method with plasmids pKS391 (*mCherry-natNT2*; a gift from Ken Sawin lab) and pT909 (*4×mCherry-natNT2*) as PCR templates. *HTB2* was tagged with CFP at its C terminus at the original gene locus using a one-step PCR method with plasmid pKT101 (*CFP-spHIS5*; EUROSCARF) as a PCR template. *SCC1* and *SMC3* were tagged with six tandem copies of HA at their C terminus at the original gene locus using a one-step PCR method with plasmid pYM15 (EUROSCARF) as a template. *HOS1* and *WPL1* (*RAD61*) were tagged with three tandem copies of mini aid tag at their C terminus by one-step PCR method, using plasmid pMK151 (*3×mini-aid-kanMX;* Kubota et al., 2013) as a template. Their protein degradation was facilitated within cells carrying rice *Oryza sativa TIR1* (*osTIR1*, expressed from *ADH1* promoter) in the presence of 0.5 mM auxin NAA (1-naphaleneacetic acid) (Nishimura et al., 2009). Strains *smc3Δ*, *scc1Δ* and *mad2Δ* were generated with a PCR-based gene deletion method (Amberg et al., 2005), using the plasmids pFA6-hphN1, pFA6-natNT2 and pFA-kanMX4 (EUROSCARF), respectively. *TetR-GFP*, *TetR-3×CFP*, *3×CFP-LacI*, P_{GAL}-*CDC20* and P_{MET3}-*CDC20* were used in our previous study (Renshaw et al., 2010), in which the original papers about these constructs are cited. Construction of *cdc15-as1*(L99G) (D'Aquino et al., 2005),

P_{GAL}-*SCC1 R180D R268D* (Uhlmann et al., 1999) and *SMC3-SCC1* fusion (Gruber et al., 2006) were previously reported. *SMC3* K112R and K113R mutations were introduced into *SMC3* and *SMC3-SCC1* fusion by site directed mutagenesis, and these constructs were integrated at *leu2* or *ura3* locus. The kinase activity of Cdc15-as1 was inhibited by adding 10 μM 1NM-PP1.

Construction of Smc3 cleavable by separase

The Nasmyth group previously constructed Smc3 whose coiled-coil region could be cleaved by TEV protease (Gruber et al., 2003). For this, they inserted a TEV recognition sequence at amino acid positions 250 and 968, where coiled-coil probability is low and the insertion is therefore less likely to disrupt the coiled-coil structure. To construct Smc3 whose coiled-coil region could be cleaved by separase (Smc3-2R), we inserted a separase recognition sequence (TSLEVGR; Sullivan et al., 2004), instead of a TEV recognition sequence, at the same 250 and 968 amino acid positions (between D250 and G251 and between D968 and F969). As a control, we also inserted a mutated separase recognition sequence (TSLEVGD) at the same sites. The *SMC3* coding sequence with the mutations, plus 350 bp upstream and 282 bp downstream sequences, were cloned into an integrative yeast vector pRS405. At the C terminus of the *SMC3* open reading frame, six tandem copies of *myc* epitope tags were inserted. The plasmid constructs were inserted at *leu2* locus in the diploid T10954 (*MATa/α SMC3/smc3Δ*) strain. Transformed diploid cells were sporulated, tetrads were dissected, and *SMC3-2R smc3Δ* and *SMC3-2D smc3Δ* haploid cells were obtained. These haploid cells showed a normal growth rate in YPAD media.

Live-cell imaging and image analysis

For live-cell imaging yeast cells were immobilized on a glass-bottom dish coated with concanavalin A, and time-lapse images were acquired and analyzed, as described previously (Kalantzaki et al., 2015; Tanaka et al., 2010): Briefly, we used DeltaVision Core and Elite microscopes (GE Healthcare Life Sciences), an UPlanSApo 100 × objective lens (Olympus; numerical aperture 1.40), SoftWoRX software (GE Healthcare Life Sciences), and CoolSnap HQ2 and Cascade II 512B CCD cameras (Photometrics) for image acquisition. At 25°C, we acquired seven to nine (0.7 μm apart) z sections, which were subsequently deconvolved, projected to two-dimensional images and analyzed with Volocity software (Improvision). For Figures, if cells drifted during live-cell imaging, the positions of frames of microscope images were adjusted so that the same cells are in middle of the frames at different time points. The time of completion of chromosome segregation was judged by disappearance of histone signals at the bud neck following their segregation; the disappearance of histone signals at the bud neck was confirmed after enhancing histone fluorescence signals on image analysis software. The completion of cytokinesis was monitored by disappearance of Myo1 at the bud neck (Wloka and Bi, 2012).

Western blotting

To detect myc-, HA-, AID-tagged proteins, acetyl-Smc3 and Cdc28, monoclonal anti-myc (9E11), monoclonal anti-HA (16B12, Covance), polyclonal anti-AID (a gift from Masato Kanemaki lab), monoclonal anti-acetyl-Smc3 (a gift from Katsu Shirahige lab; Borges et al., 2010) and polyclonal anti-Cdc28 (Santa Cruz) antibodies were used, respectively. To detect primary antibodies, HRP-conjugated secondary antibodies (Abcam, GE Healthcare) and IRDye secondary antibodies (Li-Cor) were used. The amount of proteins was quantified using software ImageLab 4.1 (Bio-Rad) after blots were scanned using the ChemiDoc imaging system (Bio-Rad).

Chromatin immunoprecipitation

Chromatin immunoprecipitation (ChIP), followed by quantitative PCR (ChIP-qPCR) and high throughput DNA sequencing (ChIP-seq), was carried out as described previously (Natsume et al., 2013) with some modification: Cells were incubated with 1% formaldehyde for overnight at 4°C for crosslink. To stop crosslink, glycine was added. Subsequently, cells were washed and lysed, followed by immunoprecipitation using anti-HA antibody (16B12) and magnetic beads (Dynabeads Protein A). For quantitative PCR following ChIP, we used Rotor-Gene 6000 (Corbett) and SYBR Green PCR kit (QIAGEN). Control cells lacking *HA* tag for *SMC1* (T11877) were also analyzed in Figure 2C and showed a low background (IP/input < 0.0003). High throughput DNA sequencing, following ChIP, was carried out at Edinburgh Genomics. The sequence results of ChIP-seq were converted to graphs (Figure S2E) using Integrative Genomic Viewer version 2.4 (y axis is set to 0–1500). To identify peaks in ChIP-seq, MACS version 2.1.0 (Feng et al., 2012) was used to call peaks directly from BAM files, with options "–gsize 12e6–mfold 3 100." For each detected peak, its position, enrichment with respect to background and statistical significance were calculated.

Chromosome spreads (chromosome fixation and immobilization)

Chromosomes were fixed and immobilized on a slide glass, immediately after cell lysis, as described previously (chromosome spreads) (Renshaw et al., 2010; Tanaka et al., 1997). Subsequently, they were processed for immunostaining using mouse monoclonal anti-acetyl-Smc3 (a gift from Shirahige lab; Borges et al., 2010) or anti-HA (16B12) antibody. The primary antibody was visualized by incubation with the secondary antibody goat anti-mouse IgG (Alexa Fluor 647). Chromosome DNA was stained with Hoechst. Spindle Pole Bodies (SPBs) were visualized by expression of Spc42 fused with mCherry. As demonstrated previously (Renshaw et al., 2010), in chromosome spreads, chromosomes showed elongation and the distance between two SPBs was enlarged, when cells proceeded from metaphase to anaphase. Cells in metaphase, in early anaphase and in late anaphase were defined as those in which SPB–SPB distance was < 2.5 μm, 2.5–5.5 μm and > 5.5 μm, respectively. The signals of Smc3-HA or acetyl-Smc3 on chromosomes were quantified using Imaris software (Bitplane).

QUANTIFICATION AND STATISTICAL ANALYSIS

Statistical analyses were carried out using Prism software (Graphpad). Methods of statistic tests are stated in each relevant figure legend. The null hypotheses in these tests were that the samples were collected randomly and independently from the same population. All *p*-values were two-tailed, and the null hypotheses were reasonably discarded when *p*-values were < 0.05.

DATA AND SOFTWARE AVAILABILITY

ChIP-seq data have been deposited in the GEO database under the accession number GEO: GSE96841. Original images used in figures have been deposited to Mendeley Data and are available at https://doi.org/10.17632/m64mz2tbvh.1.

Molecular Cell, Volume *68*

Supplemental Information

Smc3 Deacetylation by Hos1 Facilitates Efficient Dissolution of Sister Chromatid Cohesion during Early Anaphase

Shuyu Li, Zuojun Yue, and Tomoyuki U. Tanaka

Supplemental Information

Smc3 deacetylation by Hos1 facilitates efficient dissolution of sister chromatid cohesion during early anaphase

Shuyu Li, Zuojun Yue and Tomoyuki U. Tanaka

Centre for Gene Regulation and Expression, School of Life Sciences, University of Dundee, Dundee DD1 5EH, UK

Figure S1

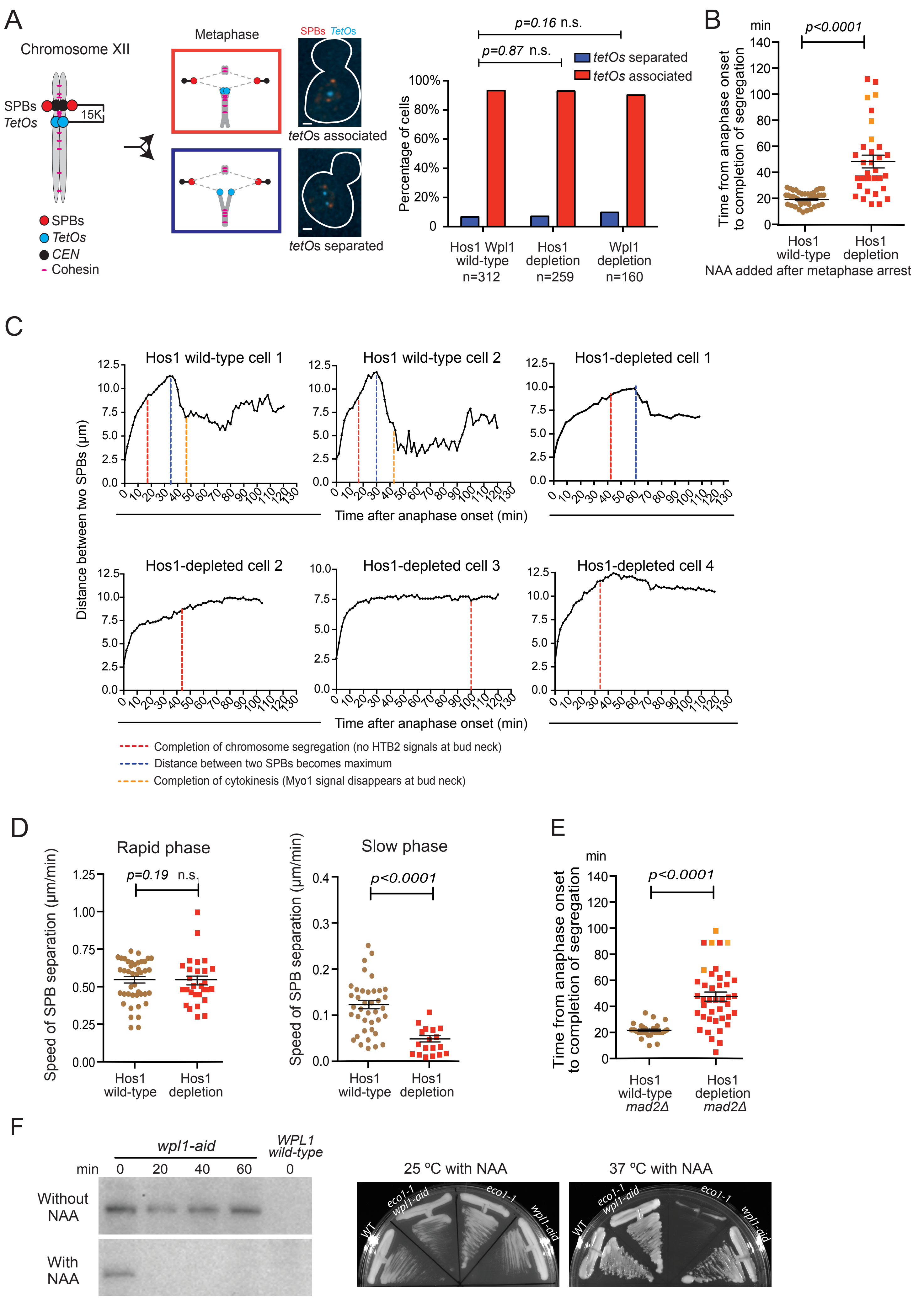
A
Chromosome XII
SPBs
TetOs
15K
SPBs
TetOs
CEN
Cohesin
Metaphase
SPBs TetOs
tetOs associated
tetOs separated
p=0.16 n.s.
p=0.87 n.s.
tetOs separated
tetOs associated
Percentage of cells
Hos1 Wpl1 wild-type n=312
Hos1 depletion n=259
Wpl1 depletion n=160
B
min
p<0.0001
Time from anaphase onset to completion of segregation
Hos1 wild-type
Hos1 depletion
NAA added after metaphase arrest
C
Hos1 wild-type cell 1
Hos1 wild-type cell 2
Hos1-depleted cell 1
Hos1-depleted cell 2
Hos1-depleted cell 3
Hos1-depleted cell 4
Distance between two SPBs (μm)
Time after anaphase onset (min)
Completion of chromosome segregation (no HTB2 signals at bud neck)
Distance between two SPBs becomes maximum
Completion of cytokinesis (Myo1 signal disappears at bud neck)
D
Rapid phase
p=0.19 n.s.
Speed of SPB separation (μm/min)
Slow phase
p<0.0001
Hos1 wild-type
Hos1 depletion
E
min
p<0.0001
Time from anaphase onset to completion of segregation
Hos1 wild-type mad2Δ
Hos1 depletion mad2Δ
F
wpl1-aid
WPL1 wild-type
min 0 20 40 60 0
Without NAA
With NAA
25 ºC with NAA
37 ºC with NAA
WT
eco1-1 wpl1-aid
eco1-1
wpl1-aid

Figure S1 (related to Figure 1)

A) After depletion of Hos1 and Wpl1, sister chromatid cohesion at peri-centromere regions is maintained as robustly as in wild-type cells
Premature sister chromatid separation around the centromere (> 10 kb from *CEN*) in metaphase is indicative of a defect in sister chromatid cohesion. We addressed whether Hos1- and Wpl-depleted cells show a defect in sister chromatid cohesion. *HOS1 WPL1* wild-type (T11773), *hos1-aid* (T11814) and *wpl1-aid* (T11804) cells with *SPC42-mCherry*, P_{MET3}-*CDC20*, TetR-*3xCFP* and *tetO*s integrated at 15 kb away from *CEN12* on chromosome XII, were arrested in G1 phase with mating pheromone in methionine-dropout medium, and subsequently released to fresh YPA medium containing 2 mM additional methionine (to deplete Cdc20) and 0.5 mM NAA (to deplete Hos1-aid and Wpl1-aid). At 2 h after release from G1, the association and separation of sister CFP dots were scored in metaphase-arrested cells (distance between two SPBs < 2 μm).
There was no significant difference in the percentage of CFP-dot separation between wild-type, Hos1- and Wpl-depleted cells. This suggests that sister chromatid cohesion remains robust in metaphase in both Hos1- and Wpl1-depleted cells.

B) Chromosome segregation in anaphase was delayed after Hos1 was depleted during metaphase arrest
HOS1 wild-type (T11556) and *hos1-aid* (T11552) cells with *HTB2-CFP*, *SPC42-mCherry*, *MYO1-mCherry* and P_{MET3}-*CDC20*, were treated with mating pheromone to arrest in G1, and released to fresh YPA medium with 2 mM additional methionine to deplete Cdc20 and to arrest in metaphase. After 1 h following release from G1, NAA was added to deplete Hos1-aid. One hour after addition of NAA, cells were transferred to methionine dropout media with NAA to release them from metaphase arrest and allow them to progress to anaphase. Subsequently time-lapse images were taken every 2 min for 2 h. Time from anaphase onset to completion of chromosome segregation was measured as in Figure 1C. Orange squares in Hos1 depletion show the time when time-lapse observation finished without chromosome segregation being completed. *p* values were obtained by *t*-test.

C) Kinetics of spindle elongation during anaphase in wild-type and Hos1-depleted cell
HOS1 wild-type (T11219) and *hos1-aid* (T11218) cells with *HTB2-CFP*, *SPC42-mCherry*, and *MYO1-mCherry* were treated and their images acquired, as in Figure 1C. The length of the spindle, i.e. the distance between two spindle pole bodies (SPBs) was measured during anaphase in representative Hos1 wild-type and Hos1-depleted cells. The anaphase onset (time 0 in graphs) is defined as the time when the distance between two SPBs reached > 2.5 μm. Red dashed lines indicate the time of completion of chromosome segregation (defined as in Figure 1C). Blue dashed lines show the time when two SPBs reached the maximum separation, and the orange dashed lines indicate the time of completion of cytokinesis (disappearance of a Myo1 ring at the bud neck; Wloka and Bi 2012).

D) Comparison of spindle elongation speed during anaphase in wild-type and Hos1-depleted cell
The image sequences acquired in C were analyzed further. The speed of SPB separation in anaphase was calculated by dividing the change in SPB–SPB distance by the time spent in a rapid phase (SPB–SPB distance changing from 2.5 to 6.5 μm) and in a slow phase (from 6.5 μm to maximum distance). *p* values were obtained by *t*-test.

E) Spindle assembly checkpoint is not involved in the delay of chromosome segregation in Hos1-depleted cells
HOS1 wild-type (T11639) and *hos1-aid* (T11640) cells with *mad2Δ*, *HTB2-CFP*, *SPC42-mCherry* and *MYO1-mCherry* were treated and their images were acquired as in Figure 1C. The time from the anaphase onset to completion of chromosome segregation was measured as in Figure 1C. *p* value was obtained by *t*-test.

F) Wpl1 was rapidly degraded and its function was abolished by the auxin-induced degron system

Left: *WPL1* wild-type (T9855) and *wpl1-aid* (T11210) cells were incubated with or without 0.5 mM NAA in asynchronous culture, and analyzed after 20, 40 and 60 min by western blotting. Wpl1-aid protein was detected with an anti-AID tag antibody. The result shows that the majority of Wpl1-aid protein was degraded within 20 min following addition of NAA.

Right: *WPL1* wild-type (T9855), *wpl-aid eco1-1* (T11815), *eco1-1* (T4107) and *wpl1-aid* (T11210) cells were inoculated on YPAD plates containing 0.5 mM NAA and grown at 25°C and 37°C for two days. After addition of auxin NAA, *wpl1-aid* suppressed the growth defects of the *eco1-1* mutant at 37°C, as did the *wpl1* deletion (Rolef Ben-Shahar et al., 2008). This suggests that Wpl1 function was abolished by the auxin-induced degron system.

Figure S2

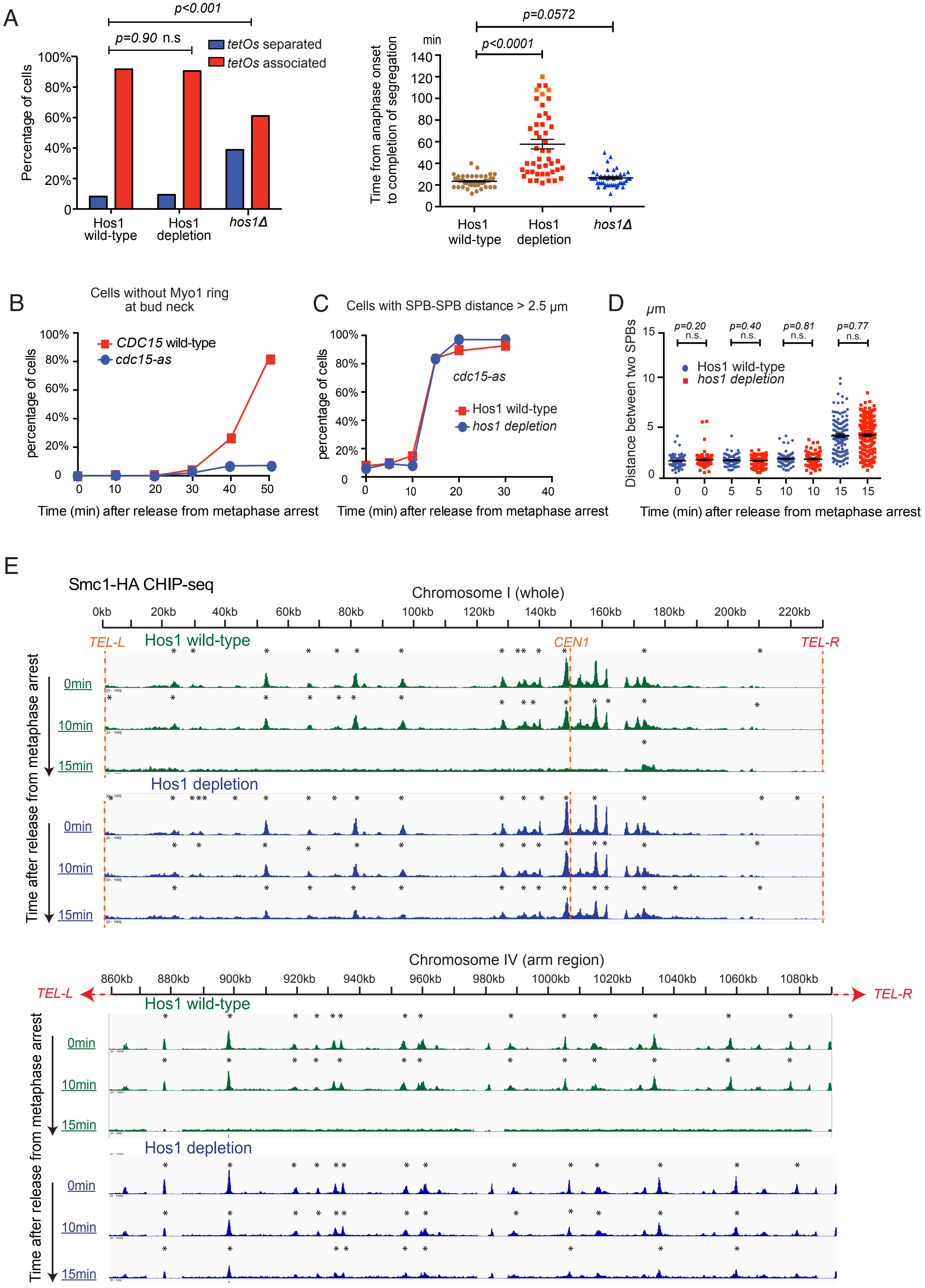
A
p<0.001
p=0.90 n.s
tetOs separated
tetOs associated
Percentage of cells
100%
80%
60%
40%
20%
0
Hos1 wild-type
Hos1 depletion
hos1Δ
p=0.0572
p<0.0001
min
Time from anaphase onset to completion of segregation
140
120
100
80
60
40
20
0
Hos1 wild-type
Hos1 depletion
hos1Δ
B
Cells without Myo1 ring at bud neck
CDC15 wild-type
cdc15-as
percentage of cells
Time (min) after release from metaphase arrest
C
Cells with SPB-SPB distance > 2.5 μm
cdc15-as
Hos1 wild-type
hos1 depletion
percentage of cells
Time (min) after release from metaphase arrest
D
μm
p=0.20 n.s.
p=0.40 n.s.
p=0.81 n.s.
p=0.77 n.s.
Hos1 wild-type
hos1 depletion
Distance between two SPBs
Time (min) after release from metaphase arrest
E
Smc1-HA CHIP-seq
Chromosome I (whole)
0kb
20kb
40kb
60kb
80kb
100kb
120kb
140kb
160kb
180kb
200kb
220kb
TEL-L
CEN1
TEL-R
Hos1 wild-type
Hos1 depletion
0min
10min
15min
Time after release from metaphase arrest
Chromosome IV (arm region)
860kb
880kb
900kb
920kb
940kb
960kb
980kb
1000kb
1020kb
1040kb
1060kb
1080kb
TEL-L
TEL-R
Hos1 wild-type
Hos1 depletion
0min
10min
15min
Time after release from metaphase arrest

Figure S2 (related to Figure 2)

A) Comparison of defects between *hos1* gene deletion (*hos1*Δ) and fresh Hos1 protein depletion

Cells with *HOS1* wild-type (T11773), *hos1-aid* (T11814) and *hos1*Δ (T13096) with *SPC42-mCherry*, P_{MET3}-*CDC20*, *TetR-3xCFP* and *tetO*s integrated at 15 kb away from *CEN12* on chromosome XII, were analyzed as in Figure S1A to compare strength of sister chromatid cohesion (left). The result suggests that, in contrast to fresh Hos1 depletion in the present cell cycle, *hos1*Δ cells show weakened cohesion, which is consistent with previous reports (Beckouet et al., 2010; Borges et al., 2010). It is thought that Smc3 is not re-cycled for the next cycle with *hos1*Δ. By contrast, cells should not have a defect in Smc3 recycling, when Hos1 is depleted following G1 phase and analyses are carried out in subsequent metaphase/anaphase. Meanwhile, in cells with *HOS1* wild-type (T11219), *hos1-aid* (T11218) and *hos1*Δ (T13109) with *HTB2-CFP*, *SPC42-mCherry*, and *MYO1-mCherry,* timing of completing chromosome segregation was analyzed as in Figure 1C (right). The result suggests that, in contrast to fresh Hos1 depletion in the present cell cycle, *hos1*Δ cells show only a marginal delay in completing chromosome segregation. We reason that, with *hos1*Δ*,* weaker cohesion in metaphase offsets a delay in cohesion removal in anaphase.

B) Inactivation of Cdc15-as kinase by an ATP analog prevents cells from completing cytokinesis

We tried to confirm that inhibiting Cdc15-as kinase by an ATP analog prevents cells from completing cytokinesis and entering the next cell cycle, in our experimental condition. *CDC15* wild-type (T12997) and *cdc15-as* (T11744) cells with *SMC1-HA, HTB2-CFP*, *SPC42-mCherry*, *MYO1-mCherry* and P_{GAL}-*CDC20* were treated, as in Figure 2C (NAA was not used). Cells were collected at 0, 10, 20, 30, 40, and 50 minutes, following release to anaphase, and fixed with 4% paraformaldehyde. At each time point, the percentage of cells without Myo1-ring signals at the bud neck was scored. Myo1 disappearance at the bud neck is an indicator of completed cytokinesis (Wloka and Bi, 2012). The result indicates that, when Cdc15-as kinase was inactivated by an ATP analog 1NM-PP1, most cells failed to complete cytokinesis.

C, D) Hos1 wild-type and Hos1-depleted cells start spindle elongation at similar timing in anaphase

HOS1 wild-type (T11875) and *hos1-aid* (T11874) cells with *cdc15-as*, *SMC1-HA*, *SPC42-mCherry, MYO1-mCherry* and P_{GAL}-*CDC20*, were treated and their images were acquired, as in Figure 2C. The SPB–SPB distance was measured in individual cells (C), and the percentage of cells with SPB–SPB distance > 2.5 μm was scored (B). Bars in C represent the mean and SE. *n.s.*: no significant difference in *t*-test.

E) ChIP-seq analysis of Smc1 localization on chromosomes.

HOS1 wild-type (T11875) and *hos1-aid* (T11874) cells (see C, D) were treated as in Figure 2C. Cells were collected at 0, 10, and 15 minutes after release to anaphase, and fixed with 1% formaldehyde. Chromatin immuno-precipitation with Smc1-HA was performed as described in STAR Methods. Immuno-precipitated DNA was analyzed using high-throughput DNA sequencing (ChIP-seq). The distribution of immuno-precipitated DNA is shown along chromosome I (whole) and chromosome IV (arm region). *TEL-L* and *TEL-R* represent the left and right telomere, respectively. Peaks in ChIP-seq were identified using MACS (Feng et al., 2012) (see STAR Methods) and those with length > 600 bp and enrichment >2.0 are highlighted by asterisks above the peaks.

Figure S3

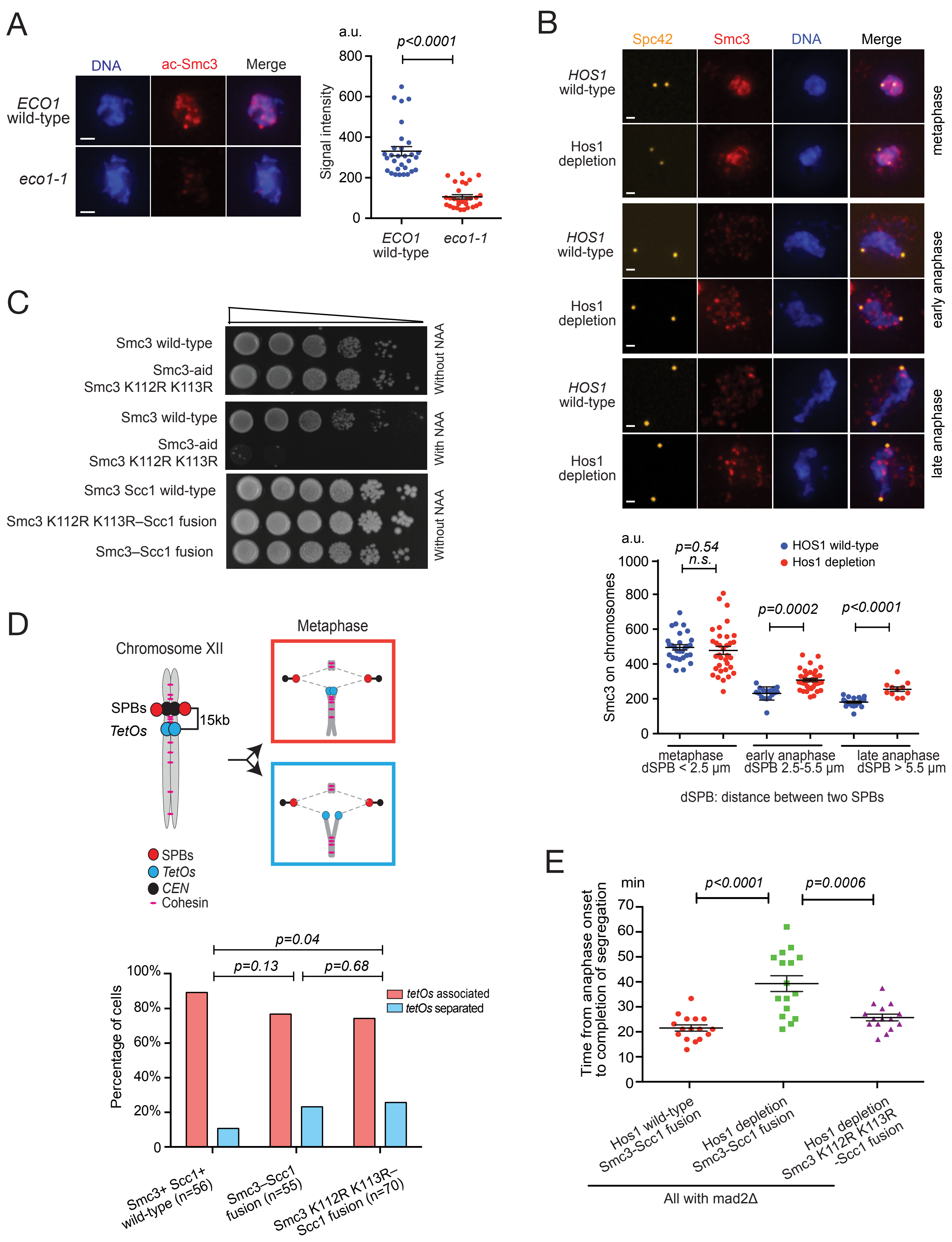

A
DNA
ac-Smc3
Merge
ECO1 wild-type
eco1-1
a.u.
p<0.0001
Signal intensity
800
600
400
200
0
ECO1 wild-type
eco1-1
B
Spc42
Smc3
DNA
Merge
HOS1 wild-type
Hos1 depletion
metaphase
early anaphase
late anaphase
a.u.
p=0.54
n.s.
p=0.0002
p<0.0001
HOS1 wild-type
Hos1 depletion
Smc3 on chromosomes
1000
800
600
400
200
0
metaphase dSPB < 2.5 µm
early anaphase dSPB 2.5-5.5 µm
late anaphase dSPB > 5.5 µm
dSPB: distance between two SPBs
C
Smc3 wild-type
Smc3-aid Smc3 K112R K113R
Without NAA
With NAA
Smc3 Scc1 wild-type
Smc3 K112R K113R–Scc1 fusion
Smc3–Scc1 fusion
D
Chromosome XII
SPBs
TetOs
15kb
Metaphase
SPBs
TetOs
CEN
Cohesin
p=0.04
p=0.13
p=0.68
Percentage of cells
100%
80%
60%
40%
20%
0
tetOs associated
tetOs separated
Smc3+ Scc1+ wild-type (n=56)
Smc3–Scc1 fusion (n=55)
Smc3 K112R K113R–Scc1 fusion (n=70)
E
min
p<0.0001
p=0.0006
Time from anaphase onset to completion of segregation
70
60
50
40
30
20
10
0
Hos1 wild-type Smc3-Scc1 fusion
Hos1 depletion Smc3-Scc1 fusion
Hos1 depletion Smc3 K112R K113R -Scc1 fusion
All with mad2Δ

Figure S3 (related to Figure 3)

A) Confirmation that a mouse anti-acetyl-Smc3 antibody can detect acetylated Smc3 on fixed and immobilized chromosomes.
ECO1 wild-type (K699) and *eco1-1* (K9435) cells were arrested in G1 phase with mating pheromone at 25°C. Then cells were incubated at 35°C for the last 30 min during G1 arrest and subsequently released from G1 arrest at 35°C by washing out mating pheromone. Cells were collected 60 min after the release. Chromosomes were fixed and immobilized on a slide glass immediately after cell lysis, followed by immunostaining of acetylated Smc3 (ac-Smc3). Scale bars represent 1 μm. *p* value was obtained by *t*-test. Bars and error bars show means and SEMs. a.u., arbitrary unit.

B) A larger amount of Smc3 remains on anaphase chromosomes in Hos1-depleted cells.
HOS1 wild type (T13179) and *hos1-aid* (T13180) cells with *SMC3-HA cdc20Δ* P_{GAL}*-CDC20 Spc42-mCherry* were treated as in Figure 3A. Chromosomes were fixed and immobilized as in Figure 3A, and Smc3-HA was stained using an anti-HA antibody. Representative cells are shown on top. The Smc3-HA signals on chromosomes were quantified (bottom). Cells in metaphase, in early anaphase and in late anaphase were defined as in Figure 3A. Scale bars represent 1 μm. *p* value was obtained by *t*-test. Bars and error bars show means and SEMs. *n.s.*, no significant difference. a.u., arbitrary unit.

C) Non-acetyl Smc3–Scc1 fusion (Smc3 K112R K113R–Scc1) can maintain cell viability in the absence of the original Smc3 and Scc1.
Ten-fold serial dilution of overnight cultures of *SMC3* wild-type (K700) and *smc3-aid smc3-K112R K113R* cells (T12989) cells were incubated for three days in the absence (top, left) and presence of (top, right) of NAA. *SMC3 SCC1* wild-type (K700), *SMC3 K112R K113R–SCC1* fusion (T12682) and *SMC3–SCC1* fusion (T12530) cells were also analyzed in the same way in the absence of NAA. In T12682 and T12530 cells, the original *SMC3* and *SCC1* genes were deleted.

D) Cells expressing non-acetyl and 'wild-type' Smc3–Scc1 fusion can similarly establish and maintain cohesion.
SMC3 SCC1 wild-type (T9968), *SMC3–SCC1* fusion (T12636) and *SMC3 K112R K113R–SCC1* fusion (T12638) cells with *SPC42-mCherry*, *TetR-GFP* and *tetO*s at 15 kb from *CEN12* on chromosome XII, were arrested in G1 phase with mating pheromone, released to fresh media and fixed at 70 min after the release. More than 70% cells were in metaphase, i.e. the distance between two SPBs was 1.5 –2.5 μm. In these cells, association and separation of sister *CEN* dots were scored (graph). *p* values were obtained by Fisher's exact test. In T12636 and T12638 cells, the original *SMC3* and *SCC1* genes were deleted.

E) Non-acetyl Smc3 mutant rescues timely chromosome segregation in Hos1-depleted cells, independently of spindle assembly checkpoint
HOS1 wild-type SMC3–SCC1 fusion (T12730), *hos1-aid SMC3–SCC1* fusion (T12729) and *hos1-aid SMC3-K112R K113R–SCC1* fusion (T12758) cells with *mad2*Δ, *SPC42-mCherry*, *MYO1-mCherry* and *HTB2-CFP*, were treated and their images acquired, as in Figure 3B. The time from the onset anaphase to completion of chromosome segregation was analyzed as in Figure 3B. The result confirms that non-acetyl Smc3-Scc1 fusion alleviates a delay in chromosome segregation in Hos1-depleted cells, and suggests that this effect is independent of spindle assembly checkpoint (in which Mad2 is a main regulator).

Figure S4

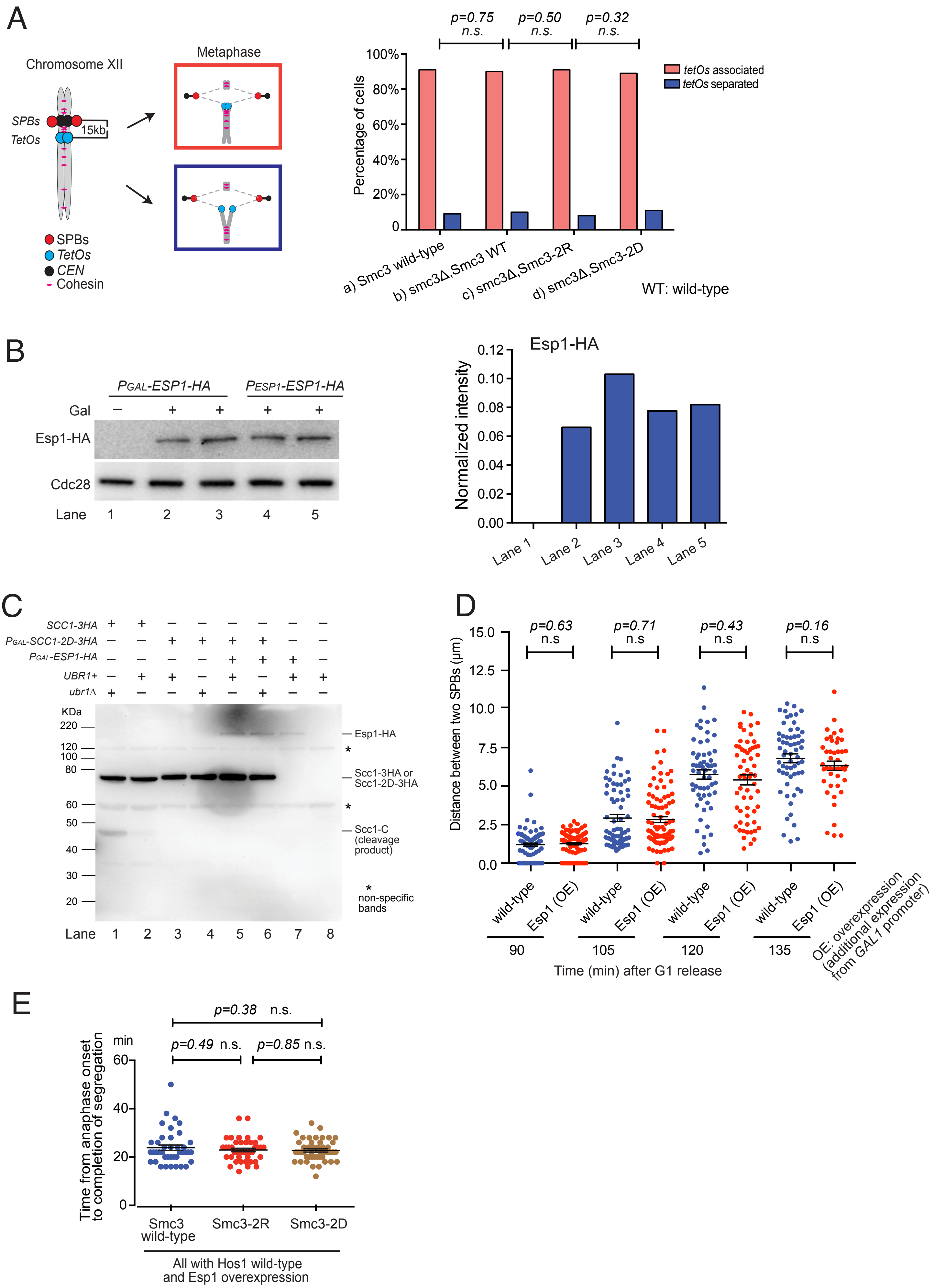
A
Chromosome XII
Metaphase
SPBs
TetOs
15kb
SPBs
TetOs
CEN
Cohesin
p=0.75 n.s.
p=0.50 n.s.
p=0.32 n.s.
Percentage of cells
100%
80%
60%
40%
20%
0
tetOs associated
tetOs separated
a) Smc3 wild-type
b) smc3Δ,Smc3 WT
c) smc3Δ,Smc3-2R
d) smc3Δ,Smc3-2D
WT: wild-type
B
PGAL-ESP1-HA
PESP1-ESP1-HA
Gal
Esp1-HA
Cdc28
Lane 1 2 3 4 5
Esp1-HA
Normalized intensity
0.12
0.10
0.08
0.06
0.04
0.02
0.00
Lane 1
Lane 2
Lane 3
Lane 4
Lane 5
C
SCC1-3HA
PGAL-SCC1-2D-3HA
PGAL-ESP1-HA
UBR1+
ubr1Δ
KDa
220
120
100
80
60
50
40
30
20
Esp1-HA
Scc1-3HA or Scc1-2D-3HA
Scc1-C (cleavage product)
* non-specific bands
Lane 1 2 3 4 5 6 7 8
D
p=0.63 n.s
p=0.71 n.s
p=0.43 n.s
p=0.16 n.s
Distance between two SPBs (μm)
15.0
12.5
10.0
7.5
5.0
2.5
0.0
wild-type
Esp1 (OE)
90
105
120
135
Time (min) after G1 release
OE: overexpression (additional expression from GAL1 promoter)
E
p=0.38 n.s.
p=0.49 n.s.
p=0.85 n.s.
Time from anaphase onset to completion of segregation
min
60
40
20
0
Smc3 wild-type
Smc3-2R
Smc3-2D
All with Hos1 wild-type and Esp1 overexpression

Figure S4 (related to Figure 4)

A) An *smc3* mutant, which can be cleaved by separase (*SMC3-2R*), and its control (*SMC3-2D*) can establish and maintain robust sister chromatid cohesion

We addressed whether *SMC3-2R* and *-2D* mutants can establish and maintain sister chromatid cohesion, similarly to *SMC3* wild-type (T12623; *b* in graph). *SMC3-2R* (T12249; *c*) and *SMC3-2D* (T12255; *d*) cells with with *SPC42-mCherry*, P_{MET3}-*CDC20*, *TetR-3xCFP* and *tetO*s integrated at 15 kb away from *CEN12* on chromosome XII, were treated and analyzed as in Figure S1A. In these cells, *SMC3* was deleted at the original locus whereas *SMC3* wild-type and mutant constructs were integrated at an auxotroph locus. Control cells with intact *SMC3* locus (T11773; *a* in graph) were also analyzed in the same way. Number of cells analyzed was 165, 245, 268 and 305 for *a*, *b*, *c* and *d*, respectively. The percentage of cells with two sister CFP dots was not significantly different among the four strains. This result indicates that *SMC3-2R* and *-2D* mutants can maintain normal robust sister chromatid cohesion in metaphase.

B) The *GAL1* and *ESP1* promoters support a similar level of separase (Esp1) expression

Cells with P_{GAL}-*ESP1-HA* (T13101) and P_{ESP1}-*ESP1-HA* (T13102), in both of which *ESP1* was fused a single copy of the *HA* tag, were cultured with (lane 2–5) and without (lane 1) galactose for 2.5 h. P_{GAL} and P_{ESP1} represent the *GAL1* and *ESP1* promoter, respectively. Lanes 2 and 3 as well as lanes 4 and 5 show the outcomes from two independent cultures of the same strain in the same culture conditions. In T13101, P_{GAL}-*ESP1-HA* was inserted at an auxotroph locus while the original *ESP1* locus was intact. In T13102, HA was fused with the *ESP1* gene at its original locus. They were analyzed by a western blot, using anti-HA and anti-Cdc28 antibodies (left). The intensity of Esp1-HA was normalized to the intensity of Cdc28 (right). The result suggests the *GAL1* and *ESP1* promoters support a similar level of Esp1 expression. In other words, in cells with P_{GAL}-*ESP1* (with the original *ESP1* locus intact), the level of Esp1 approximately doubled in the presence of galactose in medium.

C) With separase (Esp1) overexpression, Scc1-2D still does not show cleavage.

Cells with *SCC1-3HA ubr1Δ* (T13221, lane 1) and *SCC1-3HA* (T4120, lane 2) were cultured in YPAD medium. Cells with P_{GAL}-*SCC1-2D-3HA SMC3-2D* (T12201, lane 3), P_{GAL}-*SCC1-2D-3HA ubr1Δ SMC3-2D* (T13231, lane 4), P_{GAL}-*SCC1-2D-3HA* P_{GAL}-*ESP1-HA SMC3-2D* (T13210, lane 5), P_{GAL}-*SCC1-2D-3HA* P_{GAL}-*ESP1-HA ubr1Δ SMC3-2D* (T13220, lane 6) and P_{GAL}-*ESP1-HA* (T12893, lane 7) were cultured in YPA-raffinose medium overnight and subsequently in YPA-raffinose-galactose media for 2.5 h. Strain (K699) without any HA-tagged genes was used as a control (lane 8). Cells were harvested from asynchronous culture and analyzed by a western blot using an anti-HA antibody. We could detect Scc1 cleavage products when *ubr1* gene was deleted (without the N-end rule pathway; lane 1). However we could not detect cleavage products of Scc1-2D even when *ubr1* was deleted, with or without Esp1 overexpression (lanes 4 and 6).

D) With separase (Esp1) overexpression, the spindle elongation still occurs with normal kinetics during anaphase.

ESP1 wild-type cells (T12258) and cells carrying P_{GAL}-*ESP1* (T12565), both with *SPC42-mCherry, MYO1-mCherry, HTB2-CFP, hos1-aid* and *SMC3-2D*, were arrested in G1 phase with mating pheromone in YPA medium with raffinose. Cells were released to fresh YPA medium containing raffinose. Galactose was added for the last 30 min during G1 arrest and during the subsequent release from G1 (to overexpress Esp1 in T12565 cells). At indicated time points, cells were collected and fixed with 4% paraformaldehyde. The distance between two SPBs was measured in individual cells and plotted. Bars show mean and SEM. *n.s.*: no significant difference in *t*-test. The result suggests there was no precocious cohesin cleavage when Esp1 was overexpressed in this condition; if there had been precocious cohesin cleavage, we would have observed earlier spindle elongation.

Note that, with separase overexpression, we did not detect a significant change in the amount of Smc3-2R cleavage products (Figure 4B, lane 5), but Smc3-2R did show a much higher rate of chromosome segregation in the presence of Scc1-2D (Figure 4C, magenta

bars). This is explained if fresh Smc3-2R cleavage is more efficient in the presence of separase overexpression, while Smc3 cleavage products in previous cycles may remain and be detected in Figure 4B.

E) *SMC3-2R* and *SMC3-2D* do not change timing of completing chromosome segregation in Hos1 wild-type cells.

SMC3 wild-type (12986), *SMC3-2R* (T13225) and *SMC3-2D* (T13226) cells with P_{GAL}-*ESP1*, *HTB2-CFP*, *SPC42-mCherry* and *MYO1-mCherry* were arrested in G1 phase with mating pheromone in YPA-raffinose media, and released to fresh media. 2% galactose was added for the last 30 min during G1 arrest and subsequent release from G1. Images were acquired as in Figure 1C. Time from the anaphase onset to completion of chromosome segregation was measured and plotted in individual cells, as in Figure 1C.

Table S1 (related to STAR Methods)

The table shows genotypes of yeast strains used in this study. All strains used in this study are derivatives of *Saccharomyces cerevisiae* W303 (K699 and K700 from K. Nasmyth lab).

Name	Genotype
K699	*MATa ade2-1 trp1-1 leu2-3,112 his3-11,15 ura3 can1-100*
K700	*MATα ade2-1 trp1-1 leu2-3,112 his3-11,15 ura3 can1-100*
K9435	*MATα eco1-1*
T4107	*MAT α eco1-1, POL1-4×GFP::kanMX*
T4120	*MATa SCC1-3HA::HIS3 smc3Δ::HIS3 leu2::SMC3-TEVsite::LEU2 trp1::P_{GAL}-TEV::TRP1*
T9968	*MATa ade1::tetR-3×CFP::hphN1 SPC42-4×mCherry::natMX6 NIC96-4×mCherry::natMX6 tetO224 (LEU2) is integrated at +15kb from CEN12*
T9855	MAT α *ura3::P_{ADH1}-osTIR1-9×myc::URA3*
T10829	*MATa leu2::TetR-GFP::LEU2 ChrXV 326K::tetO112::kanMX ChrXV his3::tetO112::HIS3 ChrXV 1070K::tetO112::HphN1 hos1-3×mini-aid::kanMX ura3::P_{ADH1}-osTIR1-9×myc::URA3*
T10830	MATa *leu2::TetR-GFP::LEU2 ChrXV 326K::tetO112::kanMX ChrXV his3::tetO112::HIS3, ChrXV 1070K::tetO112::HphN1 ura3::P_{ADH1}-osTIR1-9×myc::URA3*
T10954	*MATa/ α SMC3/smc3 Δ ::kanMX*
T11112	*MATa leu2::TetR-GFP::LEU2 ChrXV 1070K::tetO112::HphN1 hos1-3×mini-aid::kanMX ura3::P_{ADH1}-osTIR1-9×myc::URA3 SPC42-mCherry::natNT2*
T11113	*MATa leu2::TetR-GFP::LEU2 ChrXV 1070K::tetO112::HphN1 SPC42-mCherry::natNT2*
T11116	*MAT α smc3Δ::KanMX leu2::SMC3-2R-6×myc::LEU2*
T11117	*MAT α smc3Δ::KanMX leu2::SMC3-2D-6×myc::LEU2*
T11210	*MAT α wpl1-3×mini-aid::kanMX ura3::P_{ADH1}-osTIR1-9×myc::URA3*
T11218	*MATa SPC42-mCherry::natNT2 MYO1-4×mCherry::natNT2 HTB2-CFP::spHIS5 hos1-3×mini-aid::kanMX ura3::P_{ADH1}-osTIR1-9×myc::URA3*
T11219	*MAT α SPC42-mCherry::natNT2 MYO1-4×mCherry::natNT2 HTB2-CFP::spHIS5 ura3::P_{ADH1}-osTIR1-9×myc::URA3*
T11432	*MATa wpl1-3×mini-aid::kanMX ura3::P_{ADH1}-osTIR1-9×myc::URA3 SPC42-mCherry::natNT2 MYO1-4×mCherry::natNT2 HTB2-CFP::spHIS5*
T11552	*MATa hos1-3×mini-aid::kanMX ura3::P_{ADH1}-osTIR1-9×myc::URA3 SPC42-mcherry::natNT2 MYO1-4×mCherry::natNT2 HTB2-CFP::SpHIS5, P_{MET3}-CDC20::TRP1*
T11556	*MATa ura3::P_{ADH1}-osTIR1-9×myc::URA3 SPC42-mCherry::natNT2 MYO1-4×mcherry::natNT2 HTB2-CFP::SpHIS5, P_{MET3}-CDC20::TRP1*
T11639	*MATa SPC42-mCherry::natNT2 MYO1-4×mCherry::natNT2 HTB2-CFP::spHIS5 ura3::P_{ADH1}-osTIR1-9×myc::URA3 mad2 Δ ::hphNT1*
T11640	*MATa SPC42-mCherry::natNT2 MYO1-4×mCherry::natNT2 HTB2-CFP::spHIS5 hos1-3×mini-aid::kanMX ura3::P_{ADH1}-osTIR1-9×myc::URA3 mad2 Δ ::hphNT1*
T11710	*MATa leu2::P_{URA3}-TetR-GFP::LEU2 CEN15::tetOs::kanMX, ade2::lacO256::ADE2 his3::tetOs::HIS3 trp1::P_{CUP1}-3×CFP-lacI-I12-NLS::TRP1 ura3::P_{ADH1}-osTIR1-9×myc::URA3*
T11713	*MATa leu2::P_{URA3}-TetR-GFP::LEU2 CEN15::tetOs::kanMX ade2::lacO256::ADE2 his3::tetOs::HIS3 trp1::P_{CUP1}-3×CFP-lacI-I12-NLS::TRP1 hos1-aid::KanMX ura3::P_{ADH1}-osTIR1-9×myc::URA3*
T11744	*MATa ura3::P_{ADH1}-osTIR1-9×myc::URA3 cdc20 Δ ::LEU2 trp1:: P_{GAL}-CDC20::TRP1 SMC1-6×HA::HisMX MYO1-4×mCherry::natNT2 cdc15-as1(L99G)::URA3*
T11773	*MATa P_{MET3}-CDC20::TRP1 ade1::tetR-3×CFP::hphN1 SPC42-4×mCherry::natMX6 NIC96-4×mCherry::natMX6 ura3::P_{ADH1}-osTIR1-9×myc::URA3 tetO224 (LEU2) is integrated at +15-kb from CEN12*
T11804	*MATa P_{MET3}-CDC20::TRP1 ade1::tetR-3×CFP::hphN1 SPC42-4×mCherry::natMX6 NIC96-4×mCherry::natMX6 wpl1-3xmini-aid::kanMX ura3::P_{ADH1}-osTIR1-9×myc::URA3 tetO224 (LEU2) is integrated at +15-kb from CEN12*
T11815	*MAT α eco1-1 wpl1-3xmini-aid::kanMX ura3::P_{ADH1}-osTIR1-9×myc::URA3*
T11814	*MATa P_{MET3}-CDC20::TRP1 ade1::tetR-3×CFP::HPH1 SPC42-4×mCherry::natMX6 NIC96-4×mCherry::natMX6 hos1-3×mini-aid::kanMX ura3::P_{ADH1}-osTIR1-9×myc::URA3 tetO224 (LEU2) is integrated at +15kb from CEN12*
T11874	*MATa SPC42-mCherry::natNT2 MYO1-4×mCherry::natNT2 HTB2-CFP::spHIS5 hos1-3×mini-aid::kanMX ura3::P_{ADH1}-osTIR1-9×myc::URA3 cdc20 Δ ::LEU2 trp1::P_{GAL}-CDC20::TRP1 SMC1-6×HA::HisMX cdc15-as1(L99G)::URA3*
T11875	*MATa SPC42-mCherry::natNT2 MYO1-4×mCherry::natNT2 HTB2-CFP::spHIS5 ura3::P_{ADH1}-osTIR1-9×myc::URA3 cdc20 Δ ::LEU2 trp1:: P_{GAL}-CDC20::TRP1 SMC1-6×HA::HisMX cdc15-as1(L99G)::URA3*
T11877	*MATa SPC42-mCherry::natNT2 MYO1-4×mCherry::natNT2 HTB2-CFP::spHIS5 ura3::P_{ADH1}-osTIR1-9×myc::URA3 cdc20 Δ ::LEU2 trp1:: P_{GAL}-CDC20::TRP1 cdc15-as1(L99G)::URA3*
T11912	*MATa SPC42-mCherry::natNT2 MYO1-4×mCherry::natNT2 cdc20 Δ ::LEU2 trp1::GAL-CDC20::TRP1 cdc15-as1(L99G)::URA3 hos1-3×mini-aid::kanMX ura3::P_{ADH1}-osTIR1-*

	9×myc::URA3 SCC1-6×HA::hphN1
T11914	*MATa SPC42-mCherry::natNT2 MYO1-4×mCherry::natNT2 cdc20Δ::LEU2 trp1:: P_{GAL}-CDC20::TRP1 cdc15-as1(L99G)::URA3 ura3::P_{ADH1}-osTIR1-9×myc::URA3 SCC1-6×HA::hphN1*
T12200	*MATa ura3::P_{ADH1}-osTIR1-9×myc::URA3 SPC42-mCherry::natNT2 MYO1-4×mcherry::natNT2 HTB2-CFP::SpHIS5 smc3Δ::kanMX leu2::SMC3-2R-6×myc::LEU2 ade2::P_{GAL}-scc1 R180D R268D-3HA::ADE2*
T12201	*MATa ura3::P_{ADH1}-osTIR1-9×myc::URA3 SPC42-mCherry::natNT2 MYO1-4×mcherry::natNT2 HTB2-CFP::SpHIS5 smc3Δ::kanMX leu2::SMC3-2D-6×myc::LEU2 ade2::P_{GAL}-scc1 R180D R268D-3HA::ADE2*
T12249	*MATa P_{MET3}-CDC20::TRP1 ade1::tetR-3×CFP::hphN1 SPC42-4×mCherry::natMX6 NIC96-4×mCherry::natMX6 ura3::P_{ADH1}-osTIR1-9×myc::URA3 tetO224 (LEU2) is integrated at +15-kb from CEN12 smc3Δ::kanMX, leu2::SMC3-2R-6×myc::LEU2*
T12255	*MATa P_{MET3}-CDC20::TRP1 ade1::tetR-3×CFP::hphN1 SPC42-4×mCherry::natMX6 NIC96-4×mCherry::natMX6 ura3::P_{ADH1}-osTIR1-9×myc::URA3 tetO224 (LEU2) is integrated at +15kb from CEN12 smc3Δ::kanMX, leu2::SMC3-2D-6×myc::LEU2*
T12258	*MATa SPC42-mCherry::natNT2 MYO1-4×mCherry::natNT2 HTB2-CFP::spHIS5 hos1-3×mini-aid::kanMX ura3::P_{ADH1}-osTIR1-9×myc::URA3 smc3Δ::kanMX leu2::SMC3-2D-6×myc::LEU2*
T12426	*MATa/α SMC3/smc3Δ::kanMX SCC1/scc1Δ::natNT2*
T12530	*MATα smc3Δ::KanMX scc1Δ::natNT2 ura3::SMC3-SCC1::URA3 trp1::P_{ADH1}-osTIR1-9×myc::TRP1*
T12565	*MATa SPC42-mCherry::natNT2 MYO1-4×mCherry::natNT2 HTB2-CFP::spHIS5 hos1-3×mini-aid::kanMX ura3::P_{ADH1}-osTIR1-9×myc::URA3 smc3Δ::kanMX leu2::SMC3-2D-6×myc::LEU2 trp1::$P_{GAL1-10}$-ESP1-HA::TRP1*
T12566	*MATa SPC42-mCherry::natNT2 MYO1-4×mCherry::natNT2 HTB2-CFP::spHIS5 hos1-3×mini-aid::kanMX ura3::P_{ADH1}-osTIR1-9×myc::URA3 smc3Δ::KanMX leu2::SMC3-2R-6×myc::LEU2 trp1::$P_{GAL1-10}$-ESP1-HA::TRP1*
T12623	*MATa P_{MET3}-CDC20::TRP1 ade1::tetR-3×CFP::hphN1 SPC42-4×mCherry::natMX6 NIC96-4×mCherry::natMX6 ura3::P_{ADH1}-osTIR1-9×myc::URA3 tetO224 (LEU2) is integrated at +15-kb from CEN12 smc3Δ::kanMX, leu2::SMC3::LEU2*
T12636	*MATa ade1::tetR-3×CFP::hphN1 SPC42-4×mCherry::natMX6 NIC96-4×mCherry::natMX6 tetO224 (LEU2) is integrated at +15-kb from CEN12 smc3Δ::KanMX scc1Δ::natNT2 ura3::SMC3-SCC1::URA3*
T12638	*MATa ade1::tetR-3×CFP::hphN1 SPC42-4×mCherry::natMX6 NIC96-4×mCherry::natMX6 tetO224 (LEU2) is integrated at +15-kb from CEN12 smc3Δ::KanMX scc1Δ::natNT2 ura3::SMC3 K112R K113R-SCC1::URA3*
T12665	*MATa SPC42-mCherry::natNT2, MYO1-4×mcherry::natNT2 HTB2-CFP::SpHIS5 smc3Δ::KanMX scc1Δ::natNT2 ura3::SMC3-SCC1::URA3 hos1-aid::kanMX trp1::P_{ADH1}-osTIR1-9myc::TRP1*
T12666	*MATa SPC42-mCherry::natNT2, MYO1-4×mcherry::natNT2 HTB2-CFP::SpHIS5 smc3Δ::kanMX scc1Δ::natNT2 ura3::SMC3 K112R K113R-SCC1::URA3 hos1-aid::kanMX trp1::P_{ADH1}-osTIR1-9×myc::TRP1*
T12682	*MATα smc3Δ::kanMX scc1Δ::natNT2 ura3::SMC3 K112R K113R-SCC1::URA3 trp1::PADH1-osTIR1-9×myc::TRP1*
T12684	*MATa SPC42-mCherry::natNT2, MYO1-4×mcherry::natNT2 HTB2-CFP::SpHIS5 smc3Δ::KanMX scc1Δ::natNT2 ura3::SMC3-SCC1::URA3 trp1::P_{ADH1}-osTIR1-9×myc::TRP1*
T12724	*MATa MYO1-4×mCherry::natNT2 cdc20Δ::LEU2 trp1::P_{GAL}-CDC20::TRP1 cdc15-as1(L99G)::URA3 hos1-3×mini-aid::kanMX ura3::P_{ADH1}-osTIR1-9×myc::URA3 SCC1-6×HA::hphN1 ubr1-aid::kanMX*
T12726	*MATa MYO1-4×mCherry::natNT2 cdc20Δ::LEU2 trp1::P_{GAL}-CDC20::TRP1 cdc15-as1(L99G)::URA3 ura3::P_{ADH1}-osTIR1-9×myc::URA3 SCC1-6×HA::hphN1 ubr1-aid::kanMX*
T12729	*MATa SPC42-mCherry::natNT2, MYO1-4×mcherry::natNT2 HTB2-CFP::SpHIS5 smc3Δ::KanMX Scc1Δ::natNT2 ura3::SMC3-SCC1::URA3 trp1::P_{ADH1}-osTIR1-9×myc::TRP1 hos1-aid::kanMX mad2Δ::hphN1*
T12730	*MATa SPC42-mCherry::natNT2, MYO1-4×mcherry::natNT2 HTB2-CFP::SpHIS5 smc3Δ::KanMX Scc1Δ::natNT2 ura3::SMC3-SCC1::URA3 trp1::P_{ADH1}-osTIR1-9×myc::TRP1 mad2Δ::hphN1*
T12758	*MATa SPC42-mCherry::natNT2, MYO1-4×mcherry::natNT2 HTB2-CFP::SpHIS5 smc3Δ::kanMX Scc1Δ::natNT2 ura3::smc3 K112R K113R-SCC1::URA3 trp1::P_{ADH1}-osTIR1-9×myc::TRP1 hos1-aid::kanMX mad2Δ::hphN1*
T12825	*MATa ura3::P_{ADH1}-osTIR1-9×myc::URA3 SPC42-mCherry::natNT2 MYO1-4×mcherry::natNT2 HTB2-CFP::SpHIS5 smc3Δ::KanMX leu2::SMC3-2R-6×myc::LEU2 ade2::P_{GAL}-SCC1 R180D R268D-3HA::ADE2 trp1::$P_{GAL1-10}$-ESP1-HA::TRP1*
T12827	*MATa ura3::P_{ADH1}-osTIR1-9×myc::URA3 SPC42-mCherry::natNT2 MYO1-4×mcherry::natNT2 HTB2-CFP::SpHIS5 smc3Δ::kanMX leu2::SMC3-2D-6×myc::LEU2 ade2::P_{GAL}-SCC1 R180D R268D-3HA::ADE2 trp1::$P_{GAL1-10}$-*

	ESP1-HA::TRP1
T12892	*MAT α smc3Δ::KanMX leu2::SMC3-2R-6×myc::LEU2 trp1::P$_{GAL1-10}$-ESP1-HA::TRP1*
T12893	*MAT α smc3Δ::KanMX leu2::SMC3-2D-6×myc::LEU2 trp1::P$_{GAL1-10}$-ESP1-HA::TRP1*
T12975	*MAT α smc3Δ::KanMX leu2::SMC3-6×myc::LEU2*
T12976	*MAT α smc3Δ::KanMX leu2::SMC3-6×myc::LEU2 trp1::P$_{GAL1-10}$-ESP1-HA::TRP1*
T12986	*MATa ura3::P$_{ADH1}$-osTIR1-9×myc::URA3 SPC42-mCherry::natNT2 MYO1-4×mCherry::natNT2 HTB2-CFP::SpHIS5 trp1::P$_{GAL1-10}$-ESP1-HA::TRP1*
T12989	*MAT α smc3-3×mini-aid::KanMX ura3::P$_{ADH1}$-osTIR1-9×myc::URA3 leu2::SMC3 K112R K113R::LEU2*
T12997	*MATa ura3::P$_{ADH1}$-osTIR1-9×myc::URA3 cdc20 Δ ::LEU2 trp1:: P$_{GAL}$-CDC20::TRP1 SMC1-6×HA::HisMX MYO1-4×mCherry::natNT2*
T13096	*MATα P$_{MET3}$-CDC20::TRP1 ade1::tetR-3×CFP::hphN1 SPC42-4×mCherry::natMX6 NIC96-4×mCherry::natMX6 ura3::P$_{ADH1}$-osTIR1-9×myc::URA3 tetO224 (LEU2) is integrated at +15kb from CEN12 hos1Δ::kanMX*
T13109	*MATa ura3::P$_{ADH1}$-osTIR1-9×myc::URA3 SPC42-mCherry::natNT2, MYO1-4×mCherry::natNT2 HTB2-CFP::SpHIS5 hos1Δ::kanMX*
T13101	*MATa trp1::P$_{GAL}$-ESP1-HA::TRP1*
T13102	*MATa ESP1-HA::kanMX*
T13179	*MATa SMC3-6×HA::hphN1 ura3::P$_{ADH1}$-osTIR1-9×myc::URA3 cdc20 Δ ::LEU2 trp1::P$_{GAL}$-CDC20::TRP1 SPC42-mCherry::natNT2*
T13180	*MATa SMC3-6×HA::hphN1 hos1-3×mini-aid::kanMX ura3::P$_{ADH1}$-osTIR1-9×myc::URA3 cdc20 Δ ::LEU2 trp1::P$_{GAL}$-CDC20::TRP1 SPC42-mCherry::natNT2*
T13221	*MATa SCC1-3HA::HIS3 ubr1Δ::hphN1 smc3Δ::HIS3 leu2::SMC3-TEVsite::LEU2 trp1::P$_{GAL}$-TEV::TRP1*
T13231	*MATa ura3::P$_{ADH1}$-osTIR1-9×myc::URA3 SPC42-mCherry::natNT2 MYO1-4×mcherry::natNT2 HTB2-CFP::SpHIS5 smc3Δ::kanMX leu2::SMC3-2D-6×myc::LEU2 ade2::P$_{GAL}$-SCC1 R180D R268D-3HA::ADE2*
T13210	*MAT α smc3Δ::KanMX leu2::SMC3-2D-6×myc::LEU2 trp1::P$_{GAL1-10}$-ESP1-HA::TRP1 ade2::P$_{GAL}$-SCC1 R180D R268D-3HA::ADE2*
T13220	*MAT α smc3Δ::KanMX leu2::SMC3-2D-6×myc::LEU2 trp1::P$_{GAL1-10}$-ESP1-HA::TRP1 ade2::P$_{GAL}$-SCC1 R180D R268D-3HA::ADE2 ubr1Δ::hphN1*
T13225	*MAT α ura3::P$_{ADH1}$-osTIR1-9×myc::URA3 SPC42-mCherry::natNT2, MYO1-4×mCherry::natNT2 HTB2-CFP::SpHIS5 smc3Δ::KanMX leu2::SMC3-2D-6×myc::LEU2 trp1::P$_{GAL1-10}$-ESP1-HA::TRP1*
T13226	*MAT α ura3::P$_{ADH1}$-osTIR1-9×myc::URA3 SPC42-mCherry::natNT2, MYO1-4×mcherry::natNT2 HTB2-CFP::SpHIS5 smc3Δ::KanMX leu2::SMC3-2R-6×myc::LEU2 trp1::P$_{GAL1-10}$-ESP1-HA::TRP1*
